# Dynamically reconfigurable acoustofluidic metasurface for subwavelength particle manipulation and assembly

**DOI:** 10.1038/s41467-024-55337-0

**Published:** 2025-01-15

**Authors:** Sushruta Surappa, Suraj Pavagada, Fernando Soto, Demir Akin, Charles Wei, F. Levent Degertekin, Utkan Demirci

**Affiliations:** 1 Bio-Acoustic MEMS in Medicine (BAMM) Lab, Canary Center at Stanford, Department of Radiology, School of Medicine, Stanford University, California, CA USA; 2 George W. Woodruff School of Mechanical Engineering, Georgia Institute of Technology, Atlanta, GA USA

**Keywords:** Acoustics, Fluidics, Metamaterials

## Abstract

Particle manipulation plays a pivotal role in scientific and technological domains such as materials science, physics, and the life sciences. Here, we present a dynamically reconfigurable acoustofluidic metasurface that enables precise trapping and positioning of microscale particles in fluidic environments. By harnessing acoustic-structure interaction in a passive membrane resonator array, we generate localized standing acoustic waves that can be reconfigured in real-time. The resulting radiation force allows for subwavelength manipulation and patterning of particles on the metasurface at individual and collective scales, with actuation frequencies below 2 MHz. We further demonstrate the capabilities of the reconfigurable metasurface in trapping and enriching beads and biological cells flowing in microfluidic channels, showcasing its potential in high-throughput bioanalytical applications. Our versatile and biocompatible particle manipulation platform is suitable for applications ranging from the assembly of colloidal particles to enrichment of rare cells.

## Abstract (web summary)

The authors introduce a dynamically reconfigurable acoustofluidic metasurface for microscale real-time, tunable particle manipulation, using a passive resonator array and piezoelectric transducers ( < 2 MHz). This enables subwavelength manipulation and patterning, with applications in bioanalytics.

## Introduction

Particle trapping and manipulation at microscale and nanoscale dimensions has become pivotal in numerous scientific and technological domains, spanning from particle physics to biomedicine^1–5^. These advancements are primarily driven by the growing need for precise control over particles, including biological cells, microparticles, nanoparticles and colloids, to enable a wide array of applications such as lab-on-a-chip devices, biosensing platforms, and studies of fundamental particle dynamics. To achieve efficient and versatile manipulation at these small length scales, several techniques have been developed, including optical tweezers^1,2,6^, dielectrophoresis^7,8^, magnetic traps^9,10^, and acoustics^11–14^. Among these methods, acoustofluidics has garnered significant attention due to its non-contact and label-free nature, biocompatibility, and capacity to manipulate particles over larger volumes.

Acoustofluidics harnesses the mechanical forces induced by sound waves within fluidic environments to manipulate particles and fluids. These systems typically employ Surface Acoustic Wave (SAW) or Bulk Acoustic Wave (BAW) devices to achieve precise control over particles^5^. This control is achieved using acoustic radiation force and acoustic streaming. While the radiation force offers precision and control, the magnitude of force generated is inversely proportional to both the dimensions of the fluidic channel and the wavelength of the acoustic source^15^. This inverse relationship necessitates using expensive high-frequency interdigital transducers (IDT) and sophisticated electronic systems to achieve micron-level resolution when manipulating particles^16,17^. Recent advances in additive manufacturing have given rise to innovative approaches that address these challenges, including subwavelength approaches, phononic crystals and acoustic holograms^18–24^. Metasurfaces consisting of a periodic arrangement of holes, pillars and other rigid subwavelength structures have demonstrated the capability to trap and manipulate particles at length scales that are significantly smaller than the operating wavelength of the acoustic waves. However, many of these approaches are restricted to the generation of acoustic wavefields with a fixed spatial distribution determined by the geometry of the trapping surface or hologram. Modifying the wavefield typically involves altering the geometry or employing complex actuation waveforms^25–27^. A platform enabling high-resolution and dynamic manipulation of microscale particles without relying on advanced signal processing, intricate transducers, or repeated structural modifications would offer a more accessible, user-friendly, and versatile tool for a wide range of applications.

**Fig. 1:**
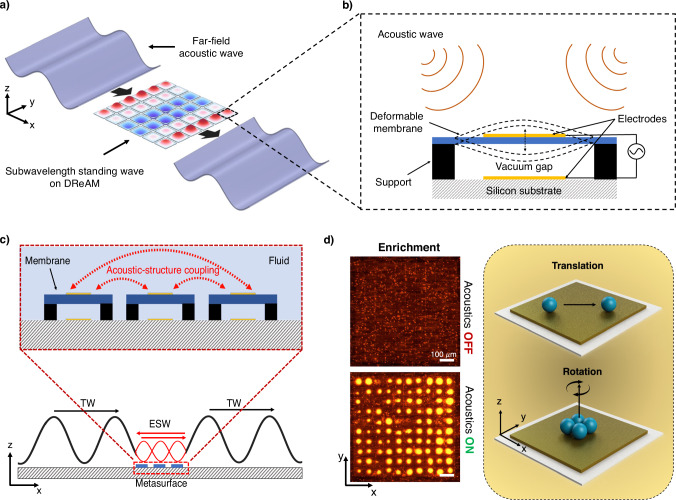
DReAM platform supporting reconfigurable evanescent standing acoustic waves for microscale particle manipulation. **a** Schematic of the membrane-based metasurface. An incoming acoustic wave excites subwavelength standing waves above DReAM. **b** Each individual resonator on the metasurface consists of a deformable, vacuum sealed membrane that vibrates when excited by an acoustic wave. **c** All the membranes on the metasurface are acoustically coupled to each other via the surrounding fluid. This results in the generation of evanescent surface waves (ESW) above DReAM when excited by a traveling acoustic wave (TW) from the far-field. Created in BioRender. Bravo, R. (2024) https://BioRender.com/e93h144. **d** The standing waves supported by DReAM generate acoustic radiation forces that can be used to trap and manipulate microscale particles at a subwavelength resolution. Colloidal crystals are formed with 5 µm fluorescent polystyrene beads on the surface of the membranes when excited by a 1 MHz acoustic wave. The findings were consistently reproduced in at least three independent experiments. Scale bars, 100 µm.

Here, we introduce a dynamically reconfigurable acoustofluidic metasurface (DReAM) for microscale trapping and manipulation in liquid environments. This passive metasurface supports multiple subwavelength acoustic wavefields, each with distinct spatial distributions and trapping positions. These distributions can be reconfigured in real-time by tuning the frequency and phase of a far-field acoustic source (Fig. 1a). DReAM is comprised of a two-dimensional array of periodically-arranged silicon micromachined membrane resonators (Fig. 1b). When a traveling acoustic wave (TW) from the far field propagates across the surface, it initiates the formation of evanescent standing waves (ESW) in the near-field above DReAM through the acoustic coupling of the resonator elements^28,29^ (Fig. 1c). We demonstrate that these reconfigurable standing wavefields generate strong acoustophoretic radiation forces capable of collectively trapping and patterning thousands of microscale particles on the metasurface (Fig. 1d). We can actively control the spatial distribution of the wavefield over DReAM, enabling either precise particle translation with a resolution of tens of micrometers or collective manipulation of monolayers of colloidal crystals. DReAM can be seamlessly integrated with a laser-cut microfluidic channel and commercial piezoelectric transducers operating in the low MHz range, enabling capture and enrichment of microscale particles and biological cells in flow. This distinctive combination of real-time reconfigurability, subwavelength resolution, high-throughput processing, biocompatibility and low operational frequency distinguishes DReAM from existing acoustofluidic manipulation methods, offering unparalleled versatility and performance.

## Results

### Design and characterization of DREAM

**Fig. 2:**
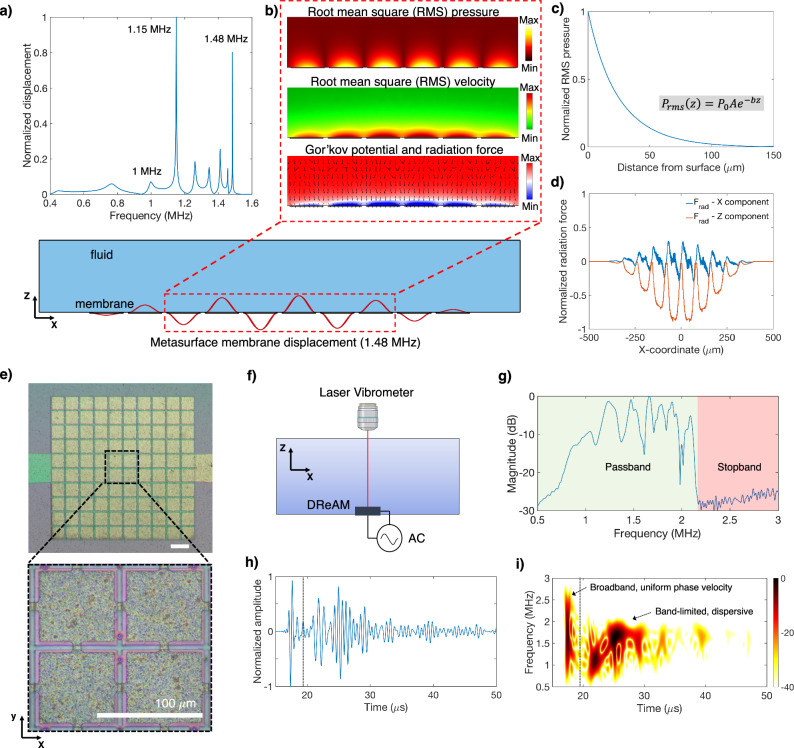
Simulation and characterization of DReAM. **a** Simulated displacement response of the center membrane reveals the presence of multiple resonances or array modes. **b** Simulated displacement, pressure, velocity and Gor’kov potential fields at 1.48 MHz. Black arrows overlaid on the Gor’kov potential field display the direction of the resultant acoustic radiation force on particles with a positive acoustic contrast factor. **c** The normalized acoustic pressure (${P}_{{rms}}$) in the Z-direction exhibits an exponential decay with increasing distance from the metasurface, characteristic of the evanescent nature of the standing wavefield. The inset equation illustrates the relationship between the root mean square (RMS) pressure and the distance from the surface (z). **d** Non-zero forces are observed only in the region above DReAM, emphasizing the local nature of the array modes. **e** Optical image of the microfabricated 10 × 10 DReAM array. A closer look at the surface shows the periodically spaced membrane resonators. Scale bar, 100 µm. **f** Schematic of the setup used to characterize DReAM. The array is excited electrically and a laser vibrometer is used to measure membrane velocity. Created in BioRender. Bravo, R. (2024) https://BioRender.com/i74r726. **g** Fourier transform of the velocity response of a single membrane on DReAM reveals multiple array resonance modes and a stopband above 2.1 MHz. Time domain response (**h**) and frequency-time transform (**i**) of the resulting excitation indicates the early arrival of low frequency components (left of dashed vertical line), subsequently followed by the slower high frequency components (right of dashed vertical line), confirming the dispersive nature of DReAM.

We employed finite element simulations to design and investigate DREAM’s dynamic behavior and characteristics. Our 2D computational model featured an array of ten periodically spaced vibrating membranes submerged in water and excited by a far-field acoustic source (Supplementary Fig. 1a). Figure 2a displays the out-of-plane displacement of the fifth membrane as a function of frequency, showcasing multiple peaks that correspond to the array’s resonance modes. These modes arise from the acoustic coupling between individual membranes and can be selectively excited by applying appropriate mode-specific excitations. Notably, some of these resonances with large quality factors (Q-factor > 100) correspond to evanescent subwavelength array modes^30^, where the local wavefield over the array exhibits a wavelength shorter than that of the incident excitation wave (Supplementary Note 1). The acoustic wavelength can be reduced by 5x to 10x over DReAM at low MHz frequencies by tuning array design parameters, such as membrane stiffness, dimensions, thickness, and pitch (see Supplementary Methods 2). To gain a deeper understanding of the surface dynamics when one of these high-Q modes was excited (f_1_ = 1.48 MHz), we analyzed the membrane displacement, acoustic pressure, particle velocity, and Gor’kov potential fields (Fig. 2b). The subwavelength nature of the excited mode is evident as alternate membranes exhibit a 180-degree out-of-phase displacement. This displacement corresponds to a standing wave on the surface of the array with a wavelength of 160 µm when excited by a far-field acoustic source with a wavelength of approximately 1 mm. The pressure and velocity fields generated by this standing wave are highly localized at the array surface and at the center of each membrane. As one moves away from the surface or approach the rigid boundary between adjacent membranes, both pressure and velocity rapidly decrease.

We calculated the acoustophoretic radiation force acting on particles in the surrounding fluid based on the Gor’kov potential field^15^. Notably, particles with a positive contrast factor experience a force directed toward the membrane’s center, coinciding with a pressure antinode. This contrasts with conventional standing-wave-based acoustofluidic systems where the radiation force typically points toward pressure nodes^31^. Conversely, particles with a negative contrast factor experience a force directed away from the membranes (Supplementary Fig. 1b). This ability to selectively attract or repel particles based on their acoustic properties provides an effective method for spatially localized particle trapping on the surface of DReAM. The evanescent nature of the wavefield is quantified in Fig. 2c, where the acoustic pressure diminishes exponentially within a wavelength’s distance from the surface. Consequently, the vertical component of the radiation force is maximum at z = 0 and rapidly reduces with increasing distance from the surface (Supplementary Fig. 1c). Figure 2d illustrates the X and Z components of the radiation force along the surface (z = 0), emphasizing that the nonzero force is localized around the resonating membranes. We also investigated the array’s response at two other resonance frequencies (f_2_ = 1.15 MHz and f_3_ = 1 MHz) and found that the wavelength of the standing wave and the strength of the resulting force field strongly depend on the specific mode that is excited (Supplementary Fig. 1d). For instance, the high-Q factor mode at f_2_ generates greater forces compared to the low-Q factor dissipative mode at f_3_ because of a tenfold greater pressure at the array surface. However, by comparing the two high-Q modes, we found that although the mode at f_2_ generates a higher surface pressure, the mode at f_1_ produces a comparable force as its shorter wavelength enables larger pressure and velocity gradients (Supplementary Fig. 1e). This versatility enables control over the spatial distribution of standing wavefields and force fields, allowing for optimization in specific applications through careful array design and excitation parameter selection.

To experimentally validate our simulation results, we fabricated a DReAM array consisting of a 10 × 10 and 20 × 20 grid of periodically arranged resonating membranes (Fig. 2e). The membranes, made of silicon nitride, were fabricated using a sacrificial release process on a silicon substrate (See methods). We characterized the vibrational response of DReAM by providing an electrical impulse excitation to the array and measuring the membrane velocity using a laser Doppler vibrometer (LDV, Fig. 2f). The frequency response (Fig. 2g) closely follows the simulation results and reveals multiple resonant peaks between 0.5 and 2 MHz, each corresponding to an array resonance mode, followed by a sharp dip beyond 2.1 MHz, indicating a stopband. We then generated a Gaussian acoustic pulse from a piezoelectric disc transducer placed adjacent to DReAM (position 1, Supplementary Fig. 2), and we measured the array response to this acoustic excitation. The time-domain response of the center membrane (Fig. 2h) shows an initial short pulse at approximately 10 µs corresponding to the broadband bulk pressure wave propagating over the array, followed by the delayed arrival of a longer, dispersive wave packet after 20 µs. A frequency-time transform (Fig. 2i) reveals that low-frequency components in this second packet precede higher-frequency waves, demonstrating the dispersive nature of DReAM, where the wave speed is a function of frequency^30^. The slower phase velocity of higher-frequency components results in shorter spatial wavelengths that are confined to the array’s surface as non-leaky evanescent waves. The time-lapse progression of the array’s response (Supplementary Video 4), visually corroborates this behavior, showcasing an initial large wavelength surface wave followed by sustained subwavelength array modes with minimal dissipation. The simulation and experimental results underscore the unique capabilities of the DReAM array, demonstrating its ability to support multiple dynamically reconfigurable evanescent standing wave modes. These results also provide a broad framework that can be applied to the design and realization of reconfigurable passive acoustic metasurfaces, and additionally, introduce an innovative approach to spatially localized particle trapping and manipulation at subwavelength scales.

### Dynamically reconfigurable patterning at subwavelength scales

**Fig. 3:**
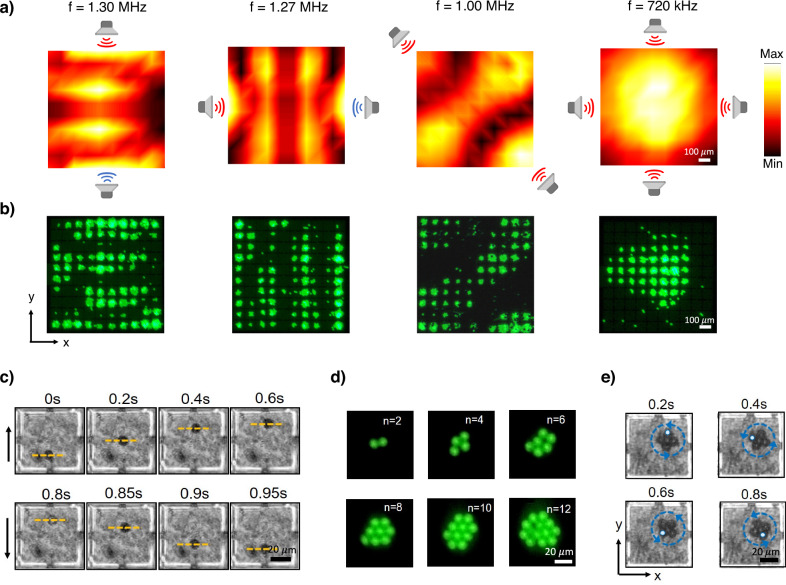
Trapping, reconfigurable patterning and manipulation of microparticles using DReAM. **a** Creation of subwavelength wavefields with distinct spatial distributions using different source configurations and emission frequencies. Scale bar, 100 µm (**b**) 10 µm fluorescent polystyrene beads align in response to the shape of the excited array mode on the metasurface. The findings were consistently reproduced in at least three independent experiments. Scale bar, 100 µm. **c** Reversible translation of a single 10 µm polystyrene bead within a single membrane of DReAM. Scale bar, 20 µm. **d** Multiple particles can be captured on a single membrane to form closely packed colloidal crystals, with n representing the number of trapped beads. Scale bar, 20 µm. **e** Crystals can be rotated on demand by modulating the frequency and amplitude of the acoustic source. Scale bar, 20 µm.

Accurate selection of frequency and source emission pattern is critical for effectively and instantly exciting various DReAM array modes from a distance. Mapping the impact of different emission patterns and frequencies on the array’s performance will allow us to determine optimal conditions for exciting specific modes. To determine the complete set of Green’s functions that connect surface displacement to emissions from each source^32^, we generated a Gaussian pulse from the piezoelectric transducer at 8 different locations around the DreAM array. We recorded the displacement of all 100 membranes with the LDV for each source location, and this allowed us to reconstruct the displacement over DReAM for any emission pattern and frequency. The reconstructed displacement field over the entire array for four different driving conditions is presented in Fig. 3a. By altering the frequency and phase of the emission source, we can excite subwavelength standing wave modes over the DReAM array with distinct field strengths and spatial distributions. This flexibility extends beyond the four modes depicted in the figure, as the array modes being orthogonal^33^ allow us to superpose a combination of field distributions on the metasurface. Iterative time-reversal techniques can also be employed to generate highly localized or focused subwavelength fields^34^. These characteristics of DReAM offers the potential to create virtually any field distribution, provided we possess prior knowledge of the Green’s function for different external emission locations as well as emissions generated by individual membranes of the array.

To visualize the standing wavefield’s impact on microscale particles, we introduced 10 μm fluorescent polystyrene beads over DReAM under the same four excitation conditions as shown in Fig. 3a. As expected, the beads were drawn towards the center of the membranes due to the radiation force and positioned themselves at the pressure antinodes (Fig. 3b). The variations observed between the reconstructed field distribution and the bead patterns can be ascribed to differing boundary conditions between the two experiments and minor deviations in the positioning of the source transducers during the experiments (Supplementary Note 2). Beads outside the area above the DReAM array did not experience this highly localized force and remained unaffected. This means that thousands of beads can be collectively manipulated and dynamically patterned by adjusting the emission source’s frequency and phase. In contrast to acoustofluidic techniques that require the Fourier synthesis of complex signals or the use of high-frequency IDTs, DReAM leverages low-frequency piezoelectric ceramic transducers (<2 MHz) to generate multiple standing wave modes, enabling efficient trapping and reconfigurable positioning of microscale particles with subwavelength resolution.

Radiation forces generated by DReAM can also be used for precise manipulation of individual beads via translation and rotation. By changing the emission frequency, we can modify the spatial pattern of the standing wave, allowing the movement of a single trapped particle to a new location on the surface (Fig. 3c). Reverting to the original emission frequency returns the particle to its initial position. We tracked the position of three adjacent beads over multiple translation cycles (Supplementary Video 1), demonstrating that particles can be repeatably and precisely positioned within a membrane with a resolution of 15 μm ($\approx$λ/100) (Supplementary Fig. 3). Using DReAM, we can also form two-dimensional (2D) colloidal crystal monolayers from the beads trapped on the membrane (Fig. 3d). The combination of in-plane primary radiation force due to the standing wave and the secondary radiation force between particles resulting from scattering creates closely packed colloidal crystals that can be rotated by adjusting the driving frequency (Supplementary Video 2 and Fig. 3e). Modification of the spatial wavefield allows for the application of an off-center force that rotates the crystal. The rotational speed depends on the wavefield’s strength and can be adjusted proportionally by varying the amplitude of the applied acoustic excitation. In essence, the ability to generate and reconfigure standing wavefields on-demand enables the trapping and manipulation of particles at both an individual and collective scale, at micrometer resolutions and low operating frequencies. This makes DReAM a valuable tool for a wide range of applications, whether they require intricate particle control or the efficient trapping of particles and colloids.

### Microparticle trapping and enrichment under flow

**Fig. 4:**
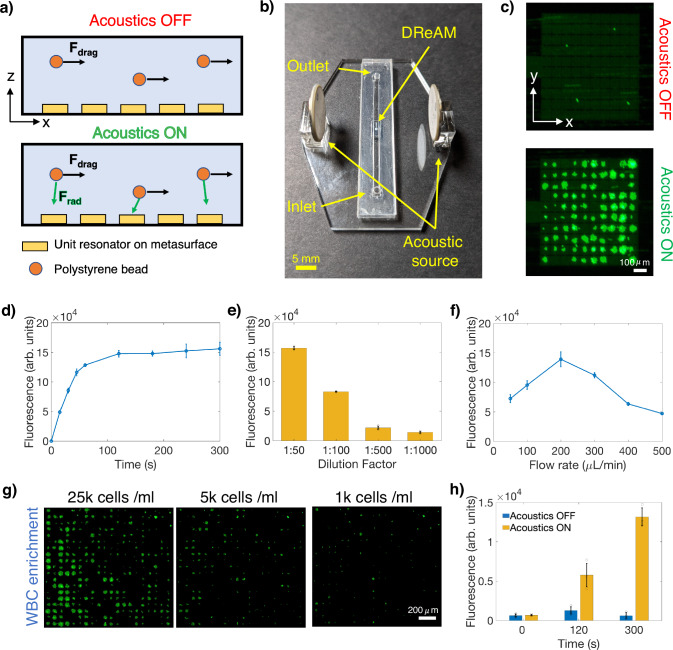
Particle enrichment in a microfluidic channel with DReAM. **a** Illustration of the different forces acting on particles in flow when the acoustics is off and on. **b** Optical image of a microfluidic platform integrating a DReAM surface and two piezoelectric acoustic sources. Scale bar, 5 mm (**c**) Particles flowing over DReAM get trapped and enriched on its surface when the acoustic source is turned on. The findings were consistently reproduced in at least three independent experiments. Scale bar, 100 µm. Characterization of the effect of different system parameters such as (**d**) capture duration (*n* = 3), (**e**) particle concentration (*n* = 3), (**f**) flow rate on the number of captured particles (*n* = 3). **g** Enrichment of WBCs at different cell concentrations. Scale bar, 200 µm (**h**) Bar graph showing the effect of acoustic enrichment on a solution containing 5000 cells/mL, measured at three time points (*n* = 3). All error bars in subplots (**d–f** and **h**) are presented as standard deviation of the mean.

Moving beyond the confines of static fluid environments, DReAM has been found to be exceptionally effective for trapping and enriching particles flowing in microfluidic channels. Two-dimensional radiation forcefields generated over the array by a single pair of source transducers enable us to counteract drag forces to trap particles in flow without requiring additional transducers or hard reflectors aligned parallel to the fluid flow axis^35^ (Fig. 4a). We mounted the planar DReAM array on the surface of a laser-cut microfluidic channel and placed two piezoelectric transducers on either side of the channel to act as the acoustic emission source (Fig. 4b). Solutions containing different concentrations of 10 µm fluorescent polystyrene beads were flown through the channel. Particles traveling over the DReAM array were trapped on the membranes when the acoustics were turned on (Fig. 4c). Over time, more particles were trapped at various sites on the array until the surface was saturated. The immobilized particles were then released by turning off the acoustic source (Supplementary Video 3). A higher trapping throughput can be realized by increasing the active area of the DReAM array (Supplementary Fig. 4a). For the same emission frequency, drive amplitude and source location, we see that the effective capture area is increased by 4 times in the case of a 20 × 20 array when compared to the 10 × 10 array. This ability to localize radiation forces based on array geometry opens the possibility to trap flowing particles in a two-dimensional plane without needing intricate channel geometries, array beamforming or multiple pairs of transducers.

We systematically varied key parameters in a series of experiments to gain an understanding of their effects on particle capture by DReAM. The input voltage to the emission source was set to the minimum value at which particle capture was observed. The polystyrene beads were circulated through the channel for 5 min at a flow rate of 200 µL/min and the number of particles captured was evaluated by measuring the fluorescence signal emitted by the trapped beads (measured in arbitrary units, Fig. 4e). We found that the number of captured particles sharply increased during the first 60–100 s of capture. After 100 seconds, the capture rate reduced, and the curve flattened due to the individual membranes of the array becoming saturated with beads. New particles that arrive at this time either fail to get trapped or replace previously trapped beads by displacing them from their trapped locations. The choice of the excited array mode also plays an important part in determining the trapping efficiency of the surface. Supplementary Fig. 4b illustrates that the mode excited at 1.6 MHz was able to capture 50% more particles than the 1.41 MHz mode for the same time and input voltage. This mode dependent efficiency can be influenced by the number of trapping points generated by the particular mode as well as the strength of the radiation force field. We tested DReAM’s efficiency for different concentrations of bead solutions and found that the number of particles captured is proportional to the solution’s concentration. DReAM exhibited efficient particle capture at concentrations as low as 1:1000, suggesting its potential for gentle, label-free particle enrichment in diluted solutions. We achieved this enrichment at flow rates greater than 100 µL/min, which demonstrates the suitability of this system for high throughput applications (Fig. 4h). We observed a drop-off in the capture area above 200 µL/min, as the drag force on the particles overcame the trapping force generated by DReAM (Supplementary Note 3). It is important to note that the strength of the radiation force field can be enhanced by providing greater power to the emission transducers. However, the influence of the viscous drag force becomes increasingly dominant, especially as the particle size reduces^36^. Despite this, we successfully demonstrated the capture of particles as small as 2 µm (Supplementary Fig. 4c).

Building on the platform’s ability to trap microscale particles, we demonstrate its capability to enrich biological material from low-concentration solutions. This addresses a significant challenge in the field, where traditional benchtop enrichment methods like filtration or centrifugation are often inadequate for efficiently processing small sample volumes or fragile cells. To evaluate the platform’s performance, we passed three different concentrations of 2 ml white blood cell (WBC) solutions through the microfluidic channel at a flow rate of 50 µL/min for 5 min. In each case, the cells were trapped and enriched at various points on the 20 × 20 array when the acoustics was turned on (Fig. 4g), with the number of trapped cells increasing proportionally with the initial concentration. Notably, even at the lowest concentration (1000 cells/mL), 100 ± 24 cells were found to be trapped within the observable array volume (0.34 μL), corresponding to a 294-fold enrichment within this localized region (see methods). Figure 4h further illustrates the impact of acoustic trapping on cell enrichment, showing a marked increase in the number of captured cells over time, with an enhancement in the fluorescence intensity within the array when the acoustic field is applied. As a result, this enables rapid observation of rare cells, even in samples that fall below the threshold for microscopic detection, thereby enhancing diagnostic capabilities^37,38^. Additionally, we successfully applied the system to capture SK-MEL-28, a skin cancer cell-line, showcasing its versatility in handling diverse cell types (Supplementary Fig. 4d). After releasing the trapped SK-MEL-28 cells, viability assessments indicated minimal impact on cell health due to acoustic exposure (Supplementary Fig. 4e), confirming that the cells remain viable for downstream analytical processing. These results collectively demonstrate the platform’s ability to locally trap and visualize cells on a grid without surface modifications or sheath flows, thus offering a straightforward approach to enrichment and analysis of biological samples at high throughputs.

## Discussion

Reconfigurable acoustic wavefields on a passive metasurface are leveraged to achieve precise manipulation of microscale particles in both static and flowing conditions at subwavelength resolutions. Current approaches for dynamically modifying wavefields in acoustofluidics rely on either tailoring waveforms to manipulate interference or using phased arrays, with control primarily originating from the acoustic source. In contrast, DReAM leverages acoustic coupling between vibrating membranes to locally modulate the acoustic impedance of the surrounding fluid, offering an innovative strategy for real-time control over the amplitude and phase distribution of the wavefield above the metasurface. This enables particle trapping and arrangement with enhanced resolution ($\approx$15 µm), increased throughput (>100 µL/min) and reduced operating frequencies (<2 MHz), improving upon existing state-of-art acoustic manipulation platforms^24,26,39,40^ (Supplementary Table 5).

Our results establish a foundation for further exploring the potential of reconfigurable, locally resonant acoustofluidic metasurfaces, enabling the development of application-driven designs. Simulations reveal flexibility in the design and realization of these metasurfaces, allowing for tunability of both the strength and spatial distribution of the generated fields. This adaptability is achieved by controlling parameters such as membrane stiffness and resonator arrangement, enabling customization for specific applications. We experimentally confirm this tunability by generating diverse wavefield configurations, which enable precise trapping, patterning, and manipulation of particles at both individual and collective scales in a reversible and dexterous manner. This ability to control and organize matter across multiple scales has significant promise for advancing the understanding of soft matter physics, enabling the creation of precisely controlled spheroids for organoid engineering, and allowing for the manipulation and analysis of individual cells in single-cell studies. Additionally, the planar profile of DReAM facilitates seamless integration with off-the-shelf transducers and microfluidic channels, allowing for capture of microscale particles in flowing conditions. We exploit this feature to gently trap and enrich different cell types from low-concentration solutions, offering a complementary method to centrifugation or other benchtop techniques, especially when handling low-volume samples.

Further optimization of array and channel design will enable particle isolation and enrichment with greater efficiencies and recovery rates. The current geometry is capable of trapping particles down to two microns, but redesigning DReAM with a smaller pitch and more compliant resonators could enable support for shorter wavelengths, potentially enabling nanoscale particle manipulation at low-MHz frequencies. The ability of DReAM to selectively attract or repel particles based on their acoustic properties can be exploited to develop complementary sorting and isolation approaches. Future developments could include individually addressable electrical control of each membrane, allowing for precise targeting and release of particles to and from specific locations on the metasurface. This capability could enable the capture, identification and selective release of target cells within heterogenous populations, paving the way for high-throughput bioanalytical applications, including sensitive detection and screening of specific cell types. Integration with optical techniques could expand the system’s analytical capabilities, enabling multimodal characterization of trapped targets. Ultimately, the system’s versatility and compatibility with low frequency piezoelectric transducers offers a cost-effective approach to the manipulation of particulate structures, paving the way for diverse applications ranging from self-assembly to point-of-care diagnostics.

## Methods

### Numerical simulations

The commercial finite element software COMSOL Multiphysics 6.1 (Burlington, MA, USA) was used for numerical simulations. The pressure acoustics module was used to compute the pressure and velocity fields in the fluid domain. The solid mechanics module was used to compute the membrane displacements. The coupling between the fluid and solid domains were facilitated by the acoustic-structure interaction module. The Gor’kov potential field and the acoustic radiation force field were derived through the postprocessing of the simulated pressure and velocity fields. The motion of particles due to the resultant radiation force was visualized using the particle tracing for fluid flow module. More details related to the equations for Gor’kov potential, acoustic radiation force as well as parameters used in our model can be found in Supplementary Methods 1 and Supplementary Table 1.

### Device fabrication

DReAM consists of an array of silicon nitride (Si_3_N_4_) membranes on a 500 µm Silicon (Si) Substrate. Each square membrane has an edge of 70 µm and a thickness of 1.5 µm and is created by a sacrificial release process. A 100 nm layer of Chromium (Cr) is deposited and patterned on the Si wafer to form the bottom electrode. This is followed by the deposition of a 400 nm thick layer of sputtered copper (Cu), that defines the thickness of the vacuum gap. The Si_3_N_4_ membrane is then deposited in two steps – first, a 700 nm thick layer of Si_3_N_4_ is deposited via Plasma Enhanced Chemical Vapor Deposition (PECVD). Etch holes are patterned and the sacrificial Cu layer in then released via wet etching to realize a free-standing membrane. Another 800 nm film of PECVD Si_3_N_4_ is subsequently deposited under vacuum conditions to seal the etch holes and to increase the thickness of the membrane to its final value of 1.5 µm. Finally, a 100 nm layer of Aluminum (Al) is deposited and patterned on top of the membranes to define the top electrode. The parameters and process are presented in more detail in Supplementary Table 2 and Supplementary Fig. 5. The individual arrays are then obtained after dicing the wafer using a wafer saw. The fabricated array was electrically characterized using an impedance analyzer (Agilent E4990A). The electrode pair could be used to actuate the array and the resonance frequency of the membranes in air was measured to be 4.39 MHz at a bias of 40 V (Supplementary Fig. 6a, b).

### DReAM characterization

The vibrational characteristics of the metasurface was characterized by mounting the array on a printed circuit board (PCB) and then immersing it in a dish filled with DI water. Care is taken to ensure that the dish is large enough to avoid overlapping reflections from the walls of the container. The two electrodes of DReAM were connected to a signal generator (Agilent 33500B) and a 100 ns, 10 V pulse was provided to excite the array. The membrane displacement was measured using a Polytec OFV-5000/534 Laser Doppler Vibrometer (Supplementary Fig. 7) and the output signal was acquired using a Picoscope 5000 series oscilloscope. The raw data was then averaged over 512 cycles and was processed using MATLAB (Mathworks, MA USA). Signal arrival times over the array as a function of frequency was obtained by performing a smoothened Pseudo Wigner-Ville distribution (SWVD) transform in MATLAB.

Acoustic characterization was performed by placing a 3.4 MHz piezoelectric disc transducer (STEMiNC SMD12T06R412WL) at 10 mm from the center of the array, at 8 different equidistant location around DReAM (Supplementary Fig. 2). A 2 cycle 1.5 MHz Sine wave burst was programmed in the signal generator and was fed to the piezoelectric transducer through an RF power amplifier (E&I 1020 L). The angle of actuation was intentionally chosen to be close to 90 degrees to efficiently excite the subwavelength modes on the array. To obtain the Green’s function, the temporal response of all 100 membranes on the array were recorded for each transducer location. To create the displacement maps of the various standing wave modes arising from different acoustic source configurations, the recorded signals for the source locations of interest were first added, after which a Fourier transform of the resultant signal was obtained. The amplitude and phase of each membrane was then plotted on a surface map for the frequency of interest to obtain the spatial distribution of the mode.

### Bead experiments

A microfluidic chip is fabricated by mounting the DReAM array inside a laser-cut cavity in a 3.5 mm thick cast acrylic substrate (McMaster Carr). The array is mounted such that the top of the metasurface is flush with the surface of the substrate. A channel with a length of 40 mm and an average width of 2.5 mm is then cut in a 150 µm thick double-sided tape (Adhesives Research Inc.) to define the microfluidic channel. One end of the cut tape is stuck to a 1.5 mm thick cast acrylic sheet and this assembly is then mounted over the base substrate and metasurface to create the DReAM platform. Inlet and outlet holes are laser-cut on the top cover and tubing is inserted and held in place using hot glue. Two 3.4 MHz piezoelectric transducers are mounted on either side of the channel to provide the acoustic emission source. To couple the acoustic emissions from the source to the fluidic channel, the entire assembly is lowered into a dish filled with deionized (DI) water (Supplementary Fig. 8). The output voltage from the signal generator was set at 60 mV.

Before loading any samples, a 0.01% Titron-X (Sigma Aldrich) solution was flown through the platform for two minutes to coat the microfluidic chamber. A stock solution of 10 µm fluorescent polystyrene beads (Spherotech Inc.), with an initial concentration of 1.819 × 10^7^ beads per milliliter (beads/mL), was divided into four separate tubes, each containing 2 milliliters (mL). The stock solution was diluted using a 0.01% Triton-X solution in DI water, resulting in final concentrations of 1:50, 1:100, 1:500, and 1:1000 in each respective tube. A Bartels micropump and pump controller were used to control the duration and rate of flow in the microfluidic channel. The capture duration was 60 s and the flowrate was 200 µL/min unless mentioned otherwise. 10 µm, 6 µm and 2 µm polystyrene beads diluted at a ratio of 1:50 were used for the experiment comparing the particle capture area for different bead sizes.

### Cell experiments

Whole blood samples from healthy donors were purchased through the Stanford University Blood Center. To isolate WBCs, whole blood was mixed with red blood cell (RBC) lysis buffer at a 1:10 ratio and incubated for 10 min to ensure complete lysis and effective WBC separation. After incubation, the mixture was centrifuged at 300 × *g* for 3 min and the resulting WBC cell pellet was collected and then resuspended in PBS for further studies. For enhanced on-chip visualization, WBCs were stained using CellTracker™ CMFDA green fluorescent probes (ThermoFisher Scientific Ltd) according to the manufacturer’s protocol. In brief, 1 × 10^6^ cells in 1 mL of solution were incubated with 1 µL of the fluorescent probe for 30 min. After staining, the cells were fixed with 4% paraformaldehyde (PFA) for 30 min, followed by rinsing with PBS, which preserved cell morphology over an extended period and retained fluorescence.

SK-MEL-28 cells were used to demonstrate the platform’s versatility in cell capture and assess cell viability post-release. The cells were cultured in Dulbecco’s Modified Eagle Medium (DMEM) supplemented with 10% FBS and 100 units/mL penicillin-streptomycin at 37 °C in a humidified atmosphere with 5% CO₂. To assess the effect of acoustics on cell viability, 25,000 cells/mL were trapped and released in triplicate (Supplementary Fig. 4d).

During all cell processing experiments, a flow rate of 50 µL/min was maintained to minimize mechanical stress and improve viability. Cell viability was assessed using the Countess™ automated cell counter with trypan blue dye, both before and after acoustic trapping and release. To release and collect the trapped cells, the acoustics were turned off, and a PBS wash was applied. Approximately 150 µL of the cell suspension was then collected from the outlet for viability testing. For analysis, 10 µL of the sample was mixed with 10 µL of 0.4% trypan blue and loaded into the automated counter (Supplementary Fig. 4e).

### Image acquisition and processing

The platform was operated under a microscope (Eclipse Ti, Nikon) using a 5x and 20x objective and a CMOS camera (Basler). Images and video were recorded using the Basler video recording software. The exposure was set to 50,000 with a gain of 15 for all obtained fluorescence images. The images were processed using ImageJ software. They were first converted to 8-bit greyscale and the thresholding feature was then set to a minimum of 36. The area of the remaining pixels was then calculated to determine the amount of captured particles for different conditions. A similar process (conversion to 8-bit followed by thresholding) was used to calculate the number of captured cells in Fig. 4g. The watershed function was used to separate closely spaced cells after thresholding, before counting the number of cells captured in 3 separate trials. The volume of the fluid column above DReAM was calculated based on the area of the metasurface (1.5 × 1.5 mm) and the height of the fluid channel (150 µm). The concentration of the trapped cells is expressed as number of cells/volume of fluid column above DReAM = 100 cells/0.34 µL **≈** 294,000 cells/ml. MtrackJ was used to track the translation of the particles.

### Reporting summary

Further information on research design is available in the Nature Portfolio Reporting Summary linked to this article.

## Supplementary information

Supplementary Information

Description of Additional Supplementary Files

Supplementary Movie 1

Supplementary Movie 2

Supplementary Movie 3

Supplementary Movie 4

Reporting Summary

Transparent Peer Review file

## Data Availability

The authors declare that all the data and code supporting the findings of this study are available within the article and supplementary materials provided with this paper. Further information is available from the corresponding author upon request.
