# Supplementary material for: Dynamically reconfigurable acoustofluidic metasurface for subwavelength particle manipulation and assembly
Source: Nat Commun. 2025 Jan 15;16:494. doi: 10.1038/s41467-024-55337-0 (PMC11736025; doi:10.1038/s41467-024-55337-0)
Supplement: Supplementary file 1 — Supplementary Information [file 41467_2024_55337_MOESM1_ESM.pdf]

Supplementary Information

Dynamically reconfigurable acoustofluidic metasurface for subwavelength particle manipulation and assembly

Sushruta Surappa[1], Suraj Pavagada[1], Fernando Soto[1], Demir Akin[1], Charles Wei[2], F.Levent Degertekin[2] and Utkan Demirci[1]

[1] *Canary Center at Stanford for Cancer Early Detection, Department of Radiology, School of Medicine, Stanford University, Palo Alto, California 94304-5427, USA*

[2] *George W. Woodruff School of Mechanical Engineering, Georgia Institute of Technology, Atlanta, Georgia 30318, USA*

*Corresponding author. Email: utkan@stanford.edu

Supplementary note 1. Analytical modelling of array modes supported by DReAM

To better understand the nature of array modes on DReAM, a simplified model can be constructed wherein each resonating membrane is described by a lumped stiffness (K) and a lumped mass (M) value. The effect of the surrounding fluid is considered in the form of a mutual radiation impedance $Z_r(\omega)$. The force balance equation solved for the membrane displacement $\{u\}$ in the frequency domain is given by,

$$[[K] + i\omega[Z_r(\omega)] - \omega^2[M]]\{x(\omega)\} = \{P(\omega)\} \quad (1)$$

$$\boldsymbol{u}(t) = Re\left[x(\omega)e^{i\omega t}\right] \quad (2)$$

Where P is the forcing per surface area. The mutual radiation impedance can be calculated by the green's function for a baffled point source in a semi-infinite fluid[1]

$$Z_r(\omega)_{mn} = \frac{i\omega\rho S}{2\pi r_{mn}} e^{-ikr_{mn}} \quad for\ m \neq n \quad (3)$$

$$Z_r(\omega)_{mn} = \rho_f c_f \left(\frac{1}{2}(ka_{eff})^2 + i\frac{8}{3\pi}(ka_{eff})\right) \quad for\ m = n \quad (4)$$

Where ρ_f and c_f are the density and speed of sound in the fluid respectively, S is the surface area of the resonating membrane, r_{mn} is the distance between any two membranes m and n, a_{eff} is the effective radius of a small piston and k is the angular wavenumber.

The homogenous solution to this system of equations can be solved by setting the forcing to zero. The quadratic eigenvalue problem is then linearized and solved by using a Taylor's expansion in a narrow frequency range as the radiation impedance is a function of ω^2. Doing so gives us n eigenvalues ω_n with each eigenvalue having an associated eigenvector $\{X_n\}$. Each eigenvalue and eigenvector pair represent the frequency and mode shape of the nth array mode. A system with loss will have complex eigenvalues and in such a case the solution can be written as

$$\text{If } \omega_n = a_n + ib_n$$

$$\{u(t)\} = Re\left[\sum_{n=1}^{N}\{c_n X_n\}e^{-b_n t}e^{ia_n t}\right] \quad (5)$$

And the quality factor of each mode is given by[2],

$$QF_n = \frac{1}{2\zeta_n} = \frac{a_n}{2b_n} \quad (6)$$

Solving these equations for a system comprising ten equally spaced membrane resonators submerged in fluid yields ten eigenvalues with their Q-factors (Supplementary table 4). Note that the eigenvalues and Q-factors calculated using the analytical technique closely align with the COMSOL simulations (fig 2a) and the experimentally obtained data (fig 2g). The variations

between the eigenfrequencies calculated using each of these techniques can be attributed to the fact that the analytical solution uses lumped parameters, assumes acoustic radiation from a baffled piston, and discards the effects of viscous losses. Likewise, the 2D domain simulated in COMSOL is infinitely long in the z-direction and uses plane wave radiation boundary conditions to model wave propagation. These simplifications also tend to overestimate the Q-factor as they fail to capture all the complex acoustic-fluid-structural interactions that occur in the experimental device.

Typically, modes with the lowest quality factors are found to possess modal wavelengths exceeding the sound wavelength in water, classifying them as highly radiative or 'leaky' modes[3]. In contrast, modes with higher quality factors are less radiative. As their wavelength shortens below that of sound in water, the wave speed on the array surface drops below the speed of sound in the fluid, resulting in the energy being confined to the array surface as evanescent waves. These evanescent modes, being poor at radiating acoustic energy, are correspondingly difficult to excite efficiently from the far-field. In our simulations and experiments, the acoustic source is positioned at a grazing angle of incidence — close to 90 degrees — to excite these less radiative modes more effectively.

Supplementary note 2. Reconstruction of pressure fields over DReAM and particle patterning

The LDV measurement of the metasurface and the subsequent recreation of the fields for different transducer configurations as shown in figure 3a were performed under free-field fluid conditions i.e. the surface was placed in a large container and filled with water. A single source transducer was placed at each of the eight positions around the metasurface (supplementary figure 2) and provided with an electrical impulse excitation. The response of all the membranes for an excitation from each of the 8 positions was recorded by the LDV. Note that all these measurements were taken on the surface without any boundaries or reflectors nearby. From this entire library of responses, we used linear superposition to reconstruct distinct field distributions that we might obtain for different source combinations and frequencies.

The bead patterning shown in figure 3b was achieved under significantly different boundary conditions. A microfluidic channel was created to introduce the beads over the surface in a controllable manner. The top and side walls of the channel immediately introduce reflections and other boundary effects that were not seen by the surface in the free-field LDV measurements. This would naturally cause the field distribution over the metasurface to be different from that generated in free-field conditions. Despite this, we see that bead patterns show qualitative (visual) agreement with the free-field LDV result, i.e., we see distinct patterns with periodicity in the horizontal (1.3 MHz), vertical direction (1.27 MHz) and diagonal directions (1 MHz) and bead concentration predominantly at the center of the surface (720 kHz). Note that the field strength is not the same in each of these cases. For example, the field strength in the 720 kHz mode is significantly weaker than the field in at 1.3 and 1.27 MHz, as it is a more radiative mode. Therefore, the trapping strength is also lower which results in sparse, unpopulated regions at the boundaries of the circular pattern.

Quantitative comparison between figure 3a and 3b are produced by calculating the correlation coefficient between corresponding LDV maps and bead patterns. The optical images in figure 3b are first converted into a 10 x 10 grid, which each grid square being assigned an average intensity value of the occupying pixels. The correlation is the calculated using the corr2 function in MATLAB. We obtain correlation coefficients of 0.54, 0.34, 0.59 and 0.77, implying a moderate to high correlation (based on the scale: < 0.3 - low, 0.3 – 0.7 - moderate and > 0.7 - high). Despite a visual match, a lower correlation coefficient can be attributed to the fact the quantitative comparison is between a figure generated from a discrete LDV displacement measurement (10 x 10 matrix) and an optical image (630x630 pixels) showing the patterning of beads on the surface. Furthermore, any variations in phase between the LDV measurements and bead patterning would strongly impact the calculated correlation coefficient.

Supplementary note 3. Calculation of Drag Force on a Bead in a Microfluidic Channel

Channel Dimensions and Flow Characteristics

The microfluidic channel has a height (h) of 150 μm, a width (w) of 2.5 mm, and a length (L) of 40 mm. The flow rate (Q) at which the drag force due to flow overcomes the acoustophoretic radiation force on the beads is > 200 μL/min, which corresponds to an average fluid velocity (v) of:

$$v = \frac{Q}{h \times w} = \frac{3.33 \times 10^{-9}}{150 \times 10^{-6} \times 2.5 \times 10^{-3}} = 0.0088\ m/s$$

Drag Force Calculation

For a spherical bead with a diameter (d) of 10 μm, the drag force (F_d) can be calculated using Stokes' law:

$$F_d = 6\pi\eta r v$$

where η is the fluid viscosity, r is the bead radius, and v is the average fluid velocity.

Using the values:

η = 0.001 Pa·s (viscosity of water at room temperature)
r = 5 μm = 0.000005 m (bead radius)
v = 0.0088 m/s (average fluid velocity)

Now we can assume that right before the trapped beads are overcome by the force of the moving fluid, the radiation force is equal in magnitude to the drag force. Therefore, the approximate acoustophoretic radiation force acting on the particles can be calculated as:

$F_{rad} \approx 6 \times 3.14 \times 0.001 \times 0.000005 \times 0.0088 \approx 829\ p\text{N}$

Supplementary methods 1. Numerical simulations using COMSOL Multiphysics

A 2D model was used in COMSOL to simulate the excitation of the array modes on DReAM. The pressure acoustics module was used to model the acoustic wave propagation in the fluid and the solid mechanics module captured the dynamic vibrations of the membrane resonators. The fluid-structure coupling was simulated using the acoustic-structure interaction module. The two edges of the vibrating membranes were fixed, the top surface was defined with an acoustic-structure BC and the bottom surface was free. For the fluid domain, the left edge was provided with a prescribed normal velocity BC. The top and right boundaries were assigned a plane wave radiation boundary condition and the remaining boundaries (which constituted the baffle around the membranes) was assigned a hard reflector BC. The entire domain was meshed using free triangular elements, where the maximum element size was set to $\lambda/5$, and λ is the wavelength of the acoustic wave in the fluid.

The study was conducted in the frequency domain, starting at 100 kHz to 2 MHz in steps of 1000 Hz to obtain the resultant pressure and velocity fields in the fluid domain, as well as the mechanical displacements of the membranes. The time averaged values of acoustic quantities can then be used to determine the Radiation force acting on a spherical particle in the fluid. For that, we first determine the Gor'kov Potential field using the following equation[4],

$$U_{gork} = \frac{4\pi a^3}{3}\left[f_1 \frac{1}{2\rho_f {c_f}^2}\langle p^2\rangle - f_2 \frac{3}{4}\rho_f \langle v^2\rangle\right] \qquad (7)$$

Where a is the radius of the particle, $\langle p^2\rangle$ and $\langle v^2\rangle$ represent the square of the RMS pressure and velocity respectively. f_1 and f_2 represent the monopole and dipole coefficients of the scatter and are given by,

$$f_1 = 1 - \frac{\kappa_p}{\kappa_f} \qquad (8)$$

$$f_2 = 2\frac{(\rho_p - \rho_f)}{2\rho_p + \rho_f} \qquad (9)$$

Where κ_p,κ_f and ρ_p, ρ_f are the compressibility and density of the particle and the fluid respectively.

The acoustophoretic radiation force acting on the particle can then be determined by calculating the negative gradient of the Gor'kov potential field i.e.,

$$\mathbf{F_{rad}} = -\nabla U_{gork} \qquad (10)$$

Supplementary methods 2. Selection of design parameters

The effect of array geometric parameters on wave propagation, such as membrane edge length and pitch, were first simulated to determine the target dimensions. A single membrane can be modelled as a thin plate clamped at the boundaries, and as such, the mass, elastic modulus and plate geometry such as edge length and thickness determine the fundamental or resonance frequency of the plate. The natural frequency of three square plates of different dimensions is shown in supplementary figure 9a. It can be seen that the resonance frequency of the plate increases as the edge length is decreased. Previous work on such resonator arrays[3] has shown that the array/cross-talk modes lie in the vicinity of the single membrane resonance frequency. It is presented in supplementary figure 9b that the choice of membrane dimensions determines the frequency range in which the dispersive behavior of the array can be observed.

The spacing between the membranes determines the shortest possible wavelength that can be supported on the array. Geometrically, the shortest wavelength corresponds to the array mode in which neighboring membranes are 180 degrees out of phase. Hence a smaller membrane pitch allows for an evanescent mode with a shorter wavelength, as seen in supplementary figure 9c. In theory, minimizing the membrane dimension and spacing would result in the array being able to support the shortest subwavelength modes on its surface. However, reducing the membrane dimensions also leads to an increase in the resonance frequency, so any advantage gained in shortening the wavelength is lost by requiring a higher external actuation frequency. In order to reliably fabricate the arrays and operate them in the 1-2 MHz range, we used membranes with dimensions of 70 μm x 70 μm and a spacing of 5 μm.

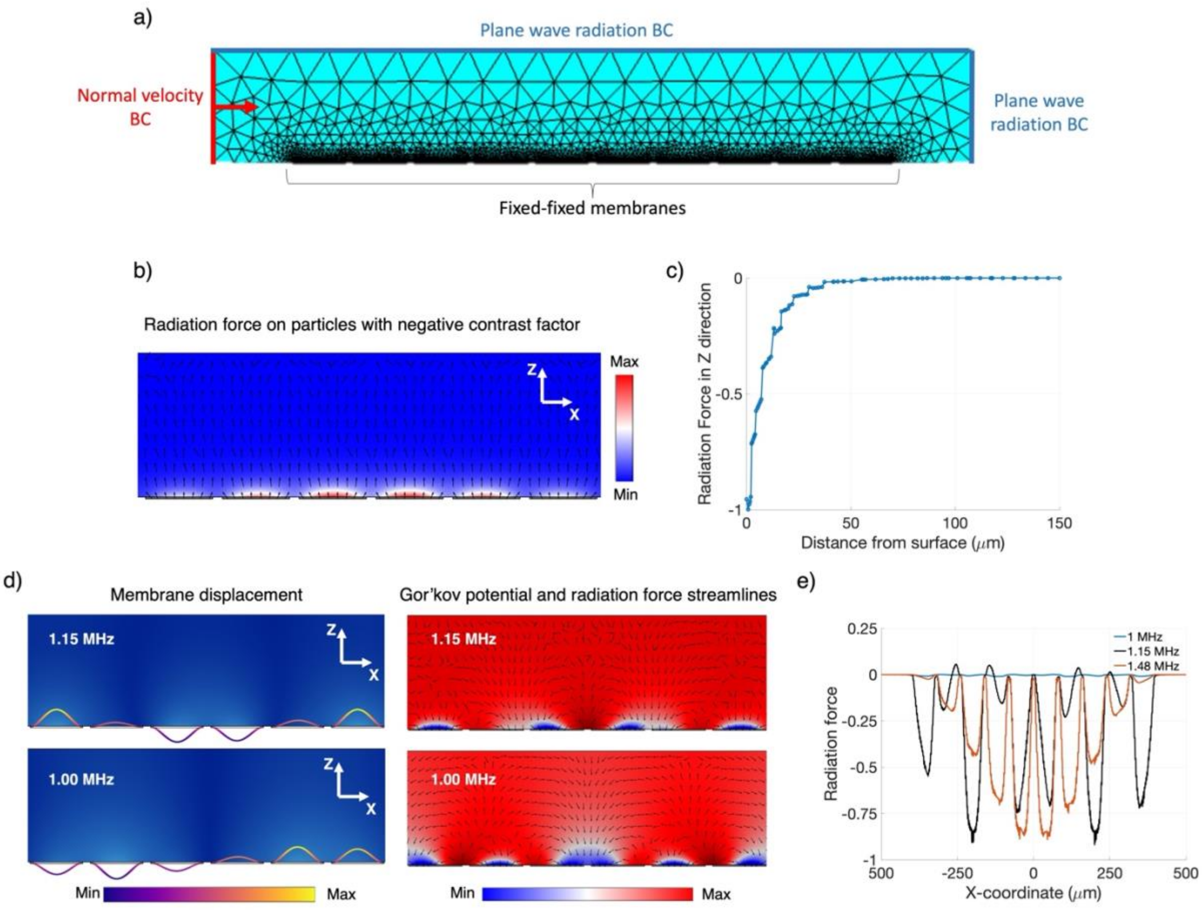

Supplementary figure 1 | COMSOL simulations. **(a)** Illustration of the the 2D FEM model used in the simulations. The domain consists of 10 membranes clamped at either end surrounded by water. The conditions at the various boundaries are marked in the figure. **(b)** Simulated Gorkov potential and radiation force field on a particle with a negative acoustic contrast factor. In this case, the force acting on the particles is directed away from the metasurface. **(c)** Magnitude of the radiation force (F) in the Z-direction reduces with distance from the metasurface due to the evanescent nature of the standing wavefield. **(d)** Simulated displacement and force fields at 1.15 MHz and 1 MHz. **(e)** Radiation force acting on a particle at z=0 for 3 different excitation frequencies.

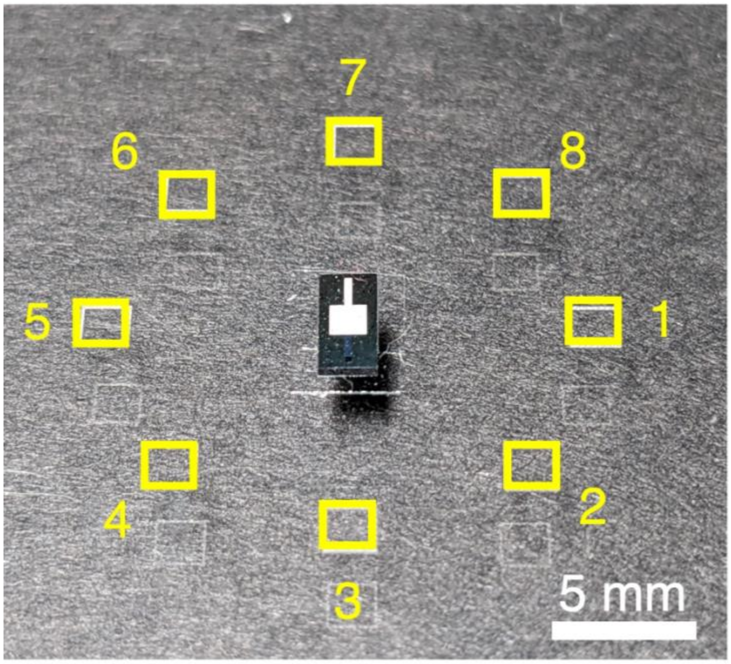

Supplementary figure 2 | Metasurface characterization. Image of the DReAM array mounted on a substrate with the surrounding yellow boxes denoting the locations where the acoustic source is placed to obtain the Green's function. Scale bar, 5 mm.

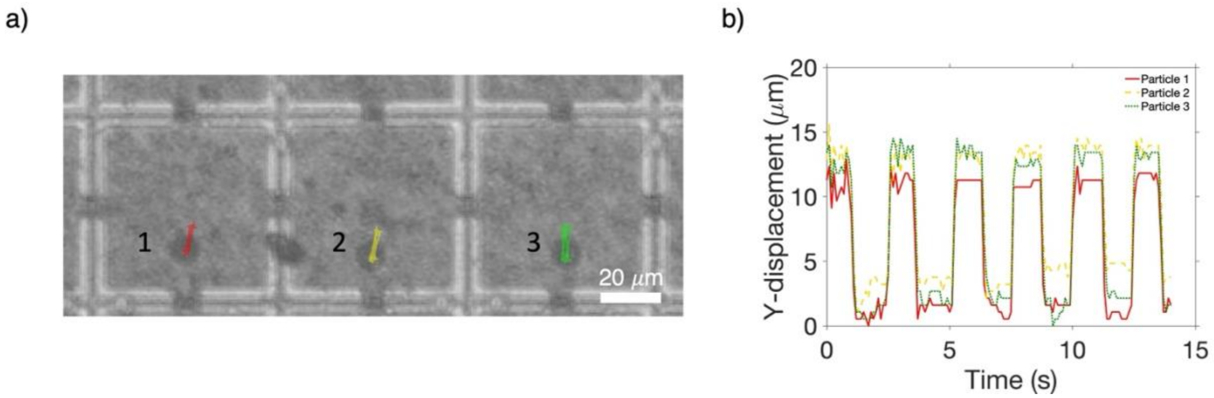

Supplementary figure 3 | Bead translation analysis. (a) Translation of 3 adjacent beads (numbered 1, 2 and 3) on DReAM when the excitation frequency is varied. The lines indicate the traces of motion of each individual bead over six up and down cycles. Scale bar, 20 μm. **(b)** The y-displacement of each bead plotted as a function of time.

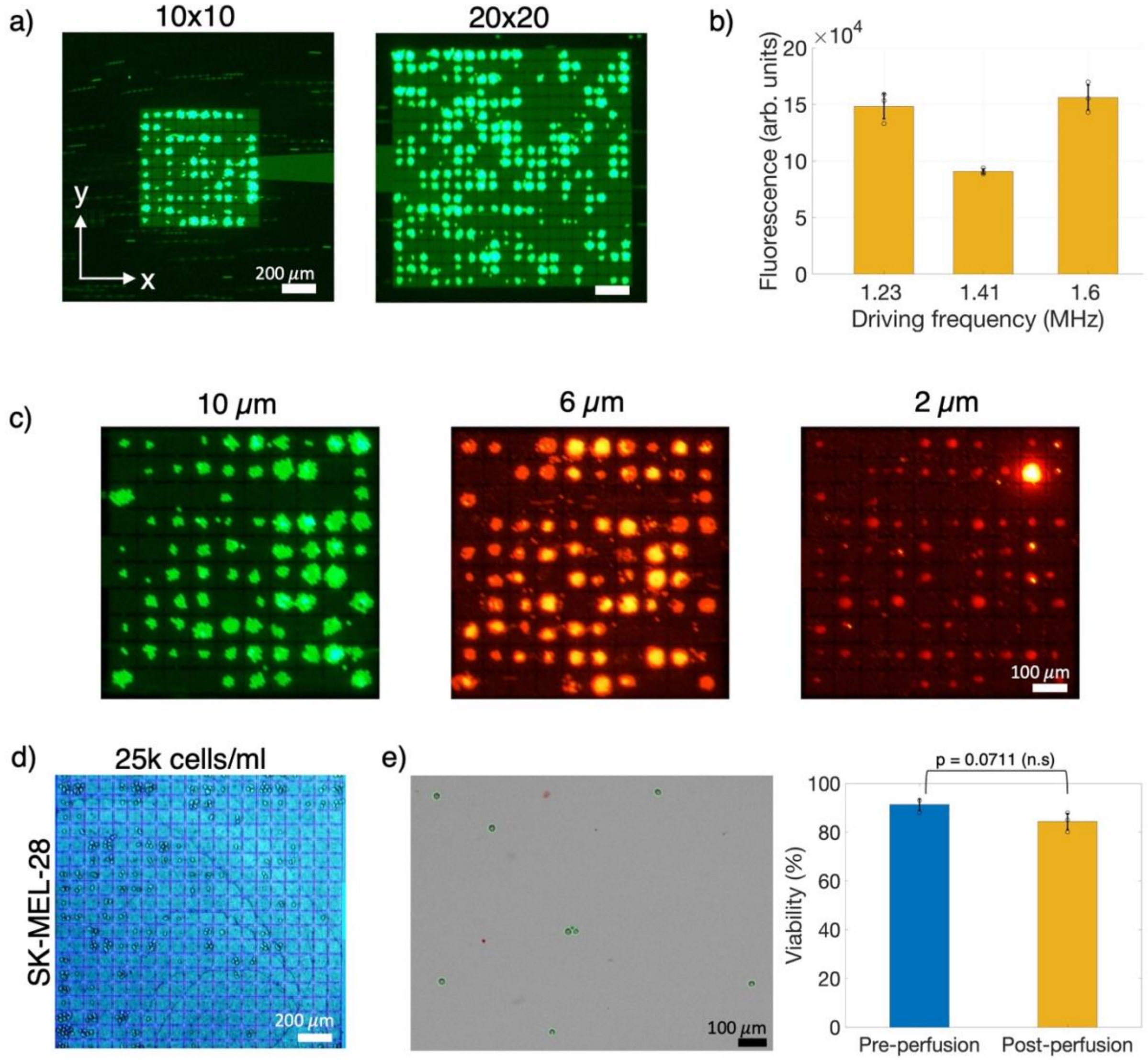

Supplementary figure 4 | Particle capture and enrichment in flow. (a) Particles captured by a 10x10 array and 20x20 array. Larger arrays can be used to capture a greater number of particles without the need to modify the channel dimensions or the frequency of the acoustic source. Scale bar, 200 µm. **(b)** Effect of acoustic driving frequency on the number of captured particles. (n=3) **(c)** Comparison of the number of trapped particles as a function of particle size. Scale bar, 100 µm. **(d)** Enrichment of SK-MEL-28 cell line from a solution containing 25,000 cells/ml on a 20x20 array. Scale bar, 200 µm. **(e)** Viability of the acoustically trapped cells after release (n=3). The viability before and after perfusion was compared for significant differences using a student's t-test (one-sided). Scale bar, 100 µm. All error bars in subplots b and e are presented as standard deviation of the mean. The findings in subplots a, c and d were consistently reproduced in at least three independent experiments.

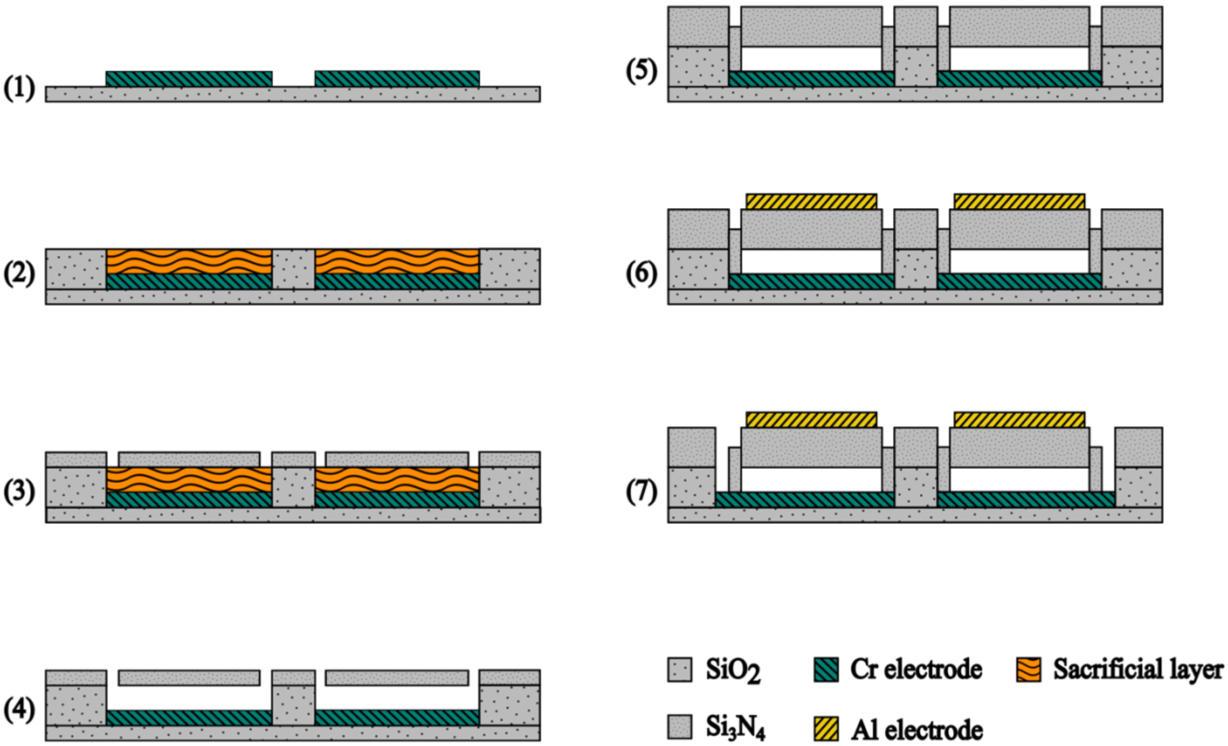

Supplementary figure 5 | Fabrication process. **(1**) Patterning of bottom electrode. **(2)** Deposition of sacrificial layer. **(3)** Deposition of initial layer of Silicon Nitride membrane and defining sacrificial etch holes. **(4)** Sacrificial layer release via wet etching. **(5)** Thickening of Silicon Nitride membrane **(6)** Deposition and patterning of top electrode **(7)** Etching down to access bottom electrode.

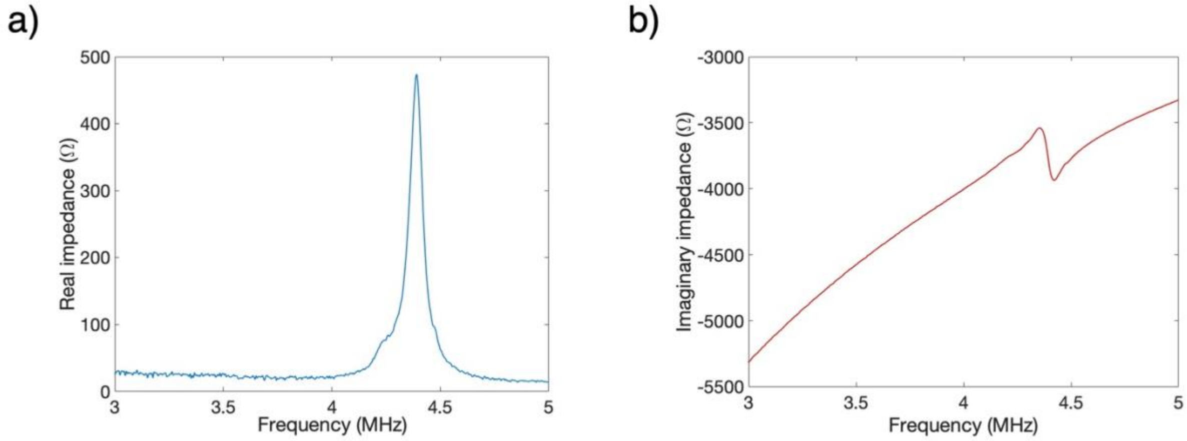

Supplementary figure 6 | Electrical characterization of metasurface. (a) Real and **(b)** imaginary impedance of the resonator array at 40V bias, as measured by an impedance analyzer.

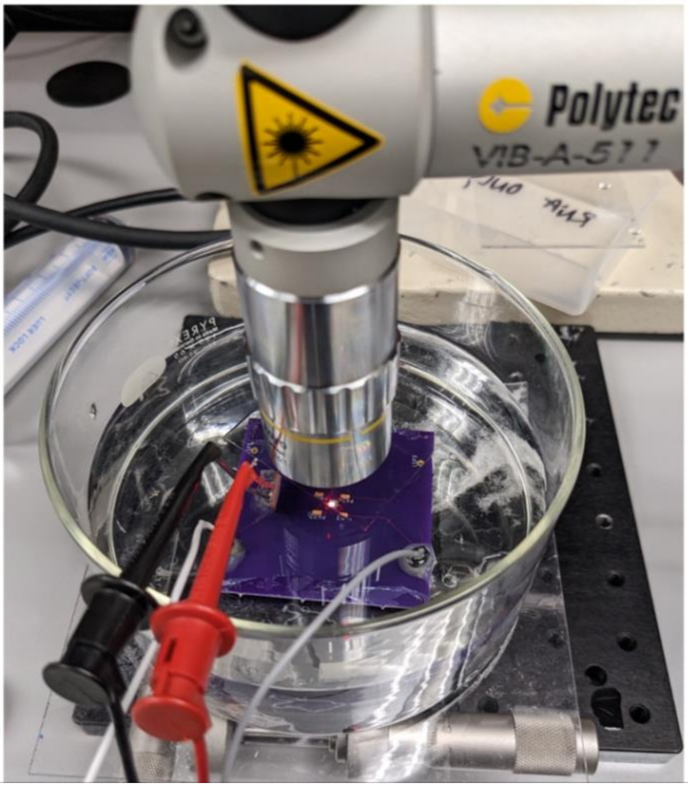

Supplementary figure 7 | LDV characterization. Photograph of the laser doppler vibrometer being used to measure the displacement of the membranes on the array.

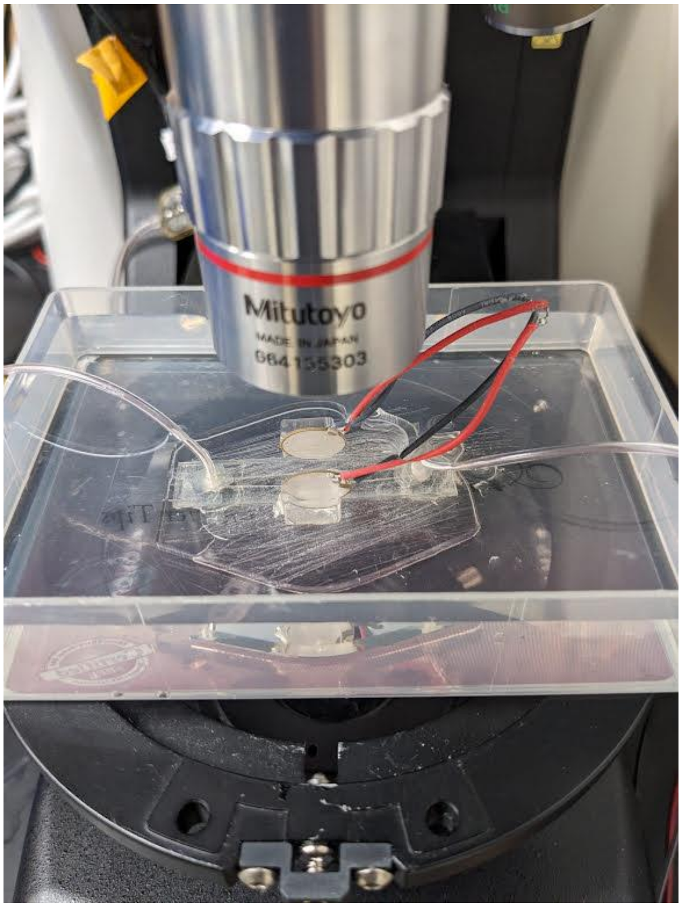

Supplementary figure 8 | Setup for microfluidic experiments. The microfluidic channel with the embedded metasurface is placed at a bottom of a tank with piezoelectric transducers mounted on either side of the channel. The tank is then filled with water to enable acoustic coupling from piezo to the fluidic channel. Pictured here is a 5x long working distance microscope objective used to image the beads on the metasurface.

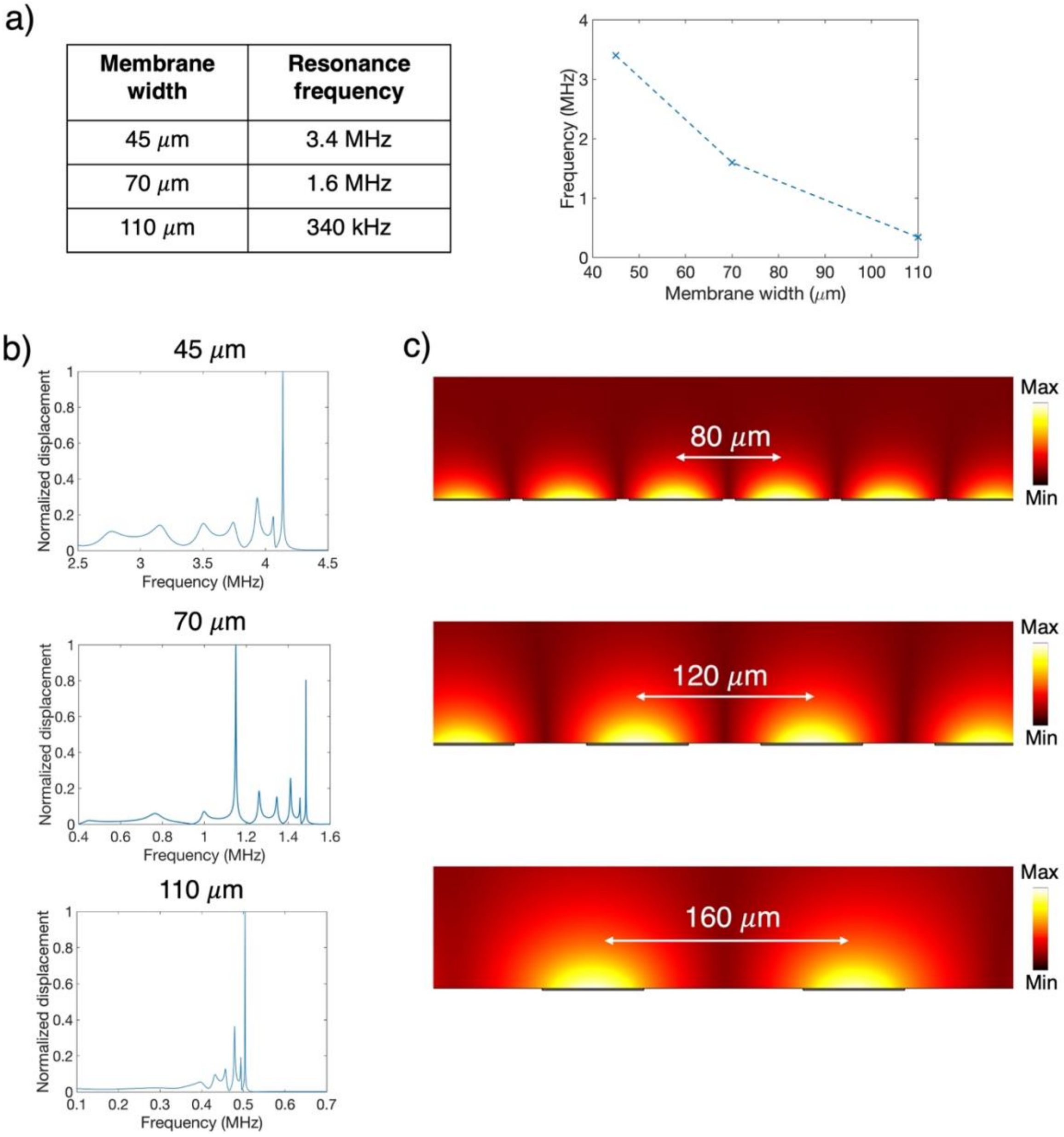

Supplementary figure 9 | Simulating the effect of membrane dimensions and spacing. (a) The resonance frequency of the membrane is a function of the membrane dimensions, with a shorter edge length resulting in a membrane with higher fundamental resonance frequency. **(b)** Arrays with membranes of different edge lengths have cross-talk modes in the vicinity of the single membrane resonance. **(c)** RMS pressure on the metasurface for three different pitch values. Increasing the spacing between membranes results in the generation of longer wavelength evanescent waves on the surface of the array. Smaller pitches result in shorter wavelengths leading to higher acoustophoretic radiation force.

Supplementary Table 1. COMSOL simulation parameters

Parameter	Value
Channel height	150 µm
Channel width	1 mm
Membrane width	70 µm
Membrane pitch	80 µm
Membrane thickness	2 µm
Membrane density	2329 Kg/m^3
Membrane young's modulus	170 x 10^9 Pa
Fluid density	1000 Kg/m^3
Speed of sound in fluid	1498 m/s
Particle size	10 µm
Particle density	1050 Kg/m^3
Particle longitudinal wave speed	2400 m/s
Particle shear wave speed	1150 m/s
Particle compressibility	1.92 x 10^{-10} Pa^{-1}

Supplementary Table 2. Effect of membrane dimensions on resonance frequency

Membrane width	Resonance frequency
45 µm	3.4 MHz
70 µm	1.6 MHz
110 µm	340 kHz

Supplementary Table 3. MEMS fabrication parameters

Parameter	Value
Gap thickness	400 nm
Membrane width	70 x 70 µm
Membrane pitch	75 µm
Membrane thickness	1.5 µm
Top Electrode width	65 x 65 µm
Top Electrode thickness	500 nm

Supplementary Table 4. Analytically calculated resonance frequencies and Q-factors

Resonance frequency	Q-factor
1.71 MHz	3
1.73 MHz	7
1.88 MHz	41
1.99 MHz	106
2.07 MHz	290
2.13 MHz	432
2.18 MHz	1086
2.22 MHz	1637
2.24 MHz	5455
2.26 MHz	6609

Supplementary Table 5. DReAM platform benchmarked against state-of-the-art acoustofluidic technologies

Published work	Operating frequency	Resolution	Reconfigura-bility	Mechanism	Sub-wavelength capability	Throughput
Our work (2024)	1 - 2 MHz	15 μm	Yes	metasurface	Yes	200 μl/min
Tayebi, 2020[5]	50 – 200 MHz	1 μm	No	metasurface	Yes	1 μL/min
Yang, 2022[6]	40 - 80 MHz	9 μm	Yes	Standing SAW	Yes	No flow
Ma, 2019[7]	5 MHz	N/A	No	Holography	No	No flow
Tung, 2019[8]	3 MHz	50 μm	No	Holography	Yes	No flow

Supplementary References

1. Kinsler, L. E., Frey, A. R., Coppens, A. B. & Sanders, J. V. *Fundamentals of Acoustics, 4th Edition*. (Wiley-VCH, 1999).
2. Ginsberg, J. H. *Mechanical and Structural Vibrations: Theory and Applications*. (Wiley, New York, 2001).
3. Lani, S., Sabra, K. G. & Degertekin, F. L. Modal and transient analysis of membrane acoustic metasurfaces. *Journal of Applied Physics* **117**, 045308 (2015).
4. Bruus, H. Acoustofluidics 7: The acoustic radiation force on small particles. *Lab on a Chip* **12**, 1014–1021 (2012).
5. Tayebi, M. *et al.* Massively Multiplexed Submicron Particle Patterning in Acoustically Driven Oscillating Nanocavities. *Small* **16**, 2000462 (2020).
6. Yang, S. *et al.* Harmonic acoustics for dynamic and selective particle manipulation. *Nat. Mater.* **21**, 540–546 (2022).
7. Ma, Z. *et al.* Acoustic Holographic Cell Patterning in a Biocompatible Hydrogel. *Advanced Materials* **32**, 1904181 (2020).
8. Tung, K.-W. *et al.* Deep, sub-wavelength acoustic patterning of complex and non-periodic shapes on soft membranes supported by air cavities. *Lab Chip* **19**, 3714–3725 (2019).
