# Supplementary material for: Dynamically reconfigurable acoustofluidic metasurface for subwavelength particle manipulation and assembly
Source: Nat Commun. 2025 Jan 15;16:494. doi: 10.1038/s41467-024-55337-0 (PMC11736025; doi:10.1038/s41467-024-55337-0)
Supplement: Supplementary file 2 — Description of Additional Supplementary Files [file 41467_2024_55337_MOESM2_ESM.pdf]

Description of Additional Supplementary Files

Supplementary Movie 1: Translation of beads.

Supplementary Movie 2: Rotation of beads.

Supplementary Movie 3: Trapping of beads on DReAM.

Supplementary Movie 4: Experimentally measured evolution of wavefield on DReAM.
