# Supplementary material for: Dynamically reconfigurable acoustofluidic metasurface for subwavelength particle manipulation and assembly
Source: Nat Commun. 2025 Jan 15;16:494. doi: 10.1038/s41467-024-55337-0 (PMC11736025; doi:10.1038/s41467-024-55337-0)
Supplement: Supplementary file 7 — Reporting Summary [file 41467_2024_55337_MOESM7_ESM.pdf]

nature portfolio

Corresponding author(s): Demirci, Utkan

Last updated by author(s): Oct 15, 2024

Reporting Summary

Nature Portfolio wishes to improve the reproducibility of the work that we publish. This form provides structure for consistency and transparency in reporting. For further information on Nature Portfolio policies, see our Editorial Policies and the Editorial Policy Checklist.

Statistics

For all statistical analyses, confirm that the following items are present in the figure legend, table legend, main text, or Methods section.

n/a	Confirmed	
☐	☒	The exact sample size (*n*) for each experimental group/condition, given as a discrete number and unit of measurement
☐	☒	A statement on whether measurements were taken from distinct samples or whether the same sample was measured repeatedly
☐	☒	The statistical test(s) used AND whether they are one- or two-sided<br>*Only common tests should be described solely by name; describe more complex techniques in the Methods section.*
☒	☐	A description of all covariates tested
☐	☒	A description of any assumptions or corrections, such as tests of normality and adjustment for multiple comparisons
☐	☒	A full description of the statistical parameters including central tendency (e.g. means) or other basic estimates (e.g. regression coefficient) AND variation (e.g. standard deviation) or associated estimates of uncertainty (e.g. confidence intervals)
☐	☒	For null hypothesis testing, the test statistic (e.g. *F*, *t*, *r*) with confidence intervals, effect sizes, degrees of freedom and *P* value noted<br>*Give P values as exact values whenever suitable.*
☒	☐	For Bayesian analysis, information on the choice of priors and Markov chain Monte Carlo settings
☒	☐	For hierarchical and complex designs, identification of the appropriate level for tests and full reporting of outcomes
☐	☒	Estimates of effect sizes (e.g. Cohen's *d*, Pearson's *r*), indicating how they were calculated

Our web collection on statistics for biologists contains articles on many of the points above.

Software and code

Policy information about availability of computer code

Data collection	Basler Video Recording Software v1.3 was used to acquire images.<br>PicoScope 6 and Matlab (R2021) was used to acquire laser doppler vibrometry data.
Data analysis	ImageJ/Fiji was used to contrast and resize images as described in the methods section.<br>ImageJ/Fiji was used to calculate the fluorescence intensity of trapped beads and count the number of trapped cells as described in the methods section.<br>MTrackJ was used to track the translation of particles as described in the methods section.<br>Matlab (R2021) was used to process and plot the acoustic field distribution over the metasurface as described in the methods section, create data plots and calculate correlation coefficient between the captured images.<br>Cell viability analysis was done using Countess 3 automated cell counter (ThermoFischer Scientific).<br>Comsol Multiphysics 6.1 was used for the acoustic simulations

For manuscripts utilizing custom algorithms or software that are central to the research but not yet described in published literature, software must be made available to editors and reviewers. We strongly encourage code deposition in a community repository (e.g. GitHub). See the Nature Portfolio guidelines for submitting code & software for further information.

Data

Policy information about availability of data

All manuscripts must include a data availability statement. This statement should provide the following information, where applicable:

- Accession codes, unique identifiers, or web links for publicly available datasets
- A description of any restrictions on data availability
- For clinical datasets or third party data, please ensure that the statement adheres to our policy

The authors declare that all the data and code supporting the findings of this study are available within the article and supplementary materials provided with this paper. Further information is available from the corresponding author upon request.

Research involving human participants, their data, or biological material

Policy information about studies with human participants or human data. See also policy information about sex, gender (identity/presentation), and sexual orientation and race, ethnicity and racism.

Reporting on sex and gender	N/A
Reporting on race, ethnicity, or other socially relevant groupings	N/A
Population characteristics	The human blood samples that we purchased were de-identified. As such, the specific characteristics such as age or health status beyond being healthy donors were not included.
Recruitment	There were no human subject recruitments. De-identified blood samples of healthy donors were purchased from the Stanford University Blood Center.
Ethics oversight	The collection of blood samples from healthy donors followed the ethics oversight protocols of the Stanford University Blood Center. The samples were purchased as deidentified.

Note that full information on the approval of the study protocol must also be provided in the manuscript.

Field-specific reporting

Please select the one below that is the best fit for your research. If you are not sure, read the appropriate sections before making your selection.

☒ Life sciences ☐ Behavioural & social sciences ☐ Ecological, evolutionary & environmental sciences

For a reference copy of the document with all sections, see nature.com/documents/nr-reporting-summary-flat.pdf

Life sciences study design

All studies must disclose on these points even when the disclosure is negative.

Sample size	For experiments involving cell enrichment, n=3 was used as the minimal independent replicate number based on traditional statistical standards. No sample size calculation was performed and this was determined to be sufficient owing to low observed variability between samples.
Data exclusions	No Data was excluded from the analysis.
Replication	All replication attempts for cell enrichment and viability were successful as the quantified values were consistent with the observations and collected images across all replicates.
Randomization	Randomization was not applicable as the study involved triplicates of a known cell type under identical conditions to assess reproducibility rather than variability between groups.
Blinding	Blinding was not relevant as the study involved a known cell type with standardized conditions.

Reporting for specific materials, systems and methods

We require information from authors about some types of materials, experimental systems and methods used in many studies. Here, indicate whether each material, system or method listed is relevant to your study. If you are not sure if a list item applies to your research, read the appropriate section before selecting a response.

Materials & experimental systems

n/a	Involved in the study	
☒	☐	Antibodies
☐	☒	Eukaryotic cell lines
☒	☐	Palaeontology and archaeology
☒	☐	Animals and other organisms
☒	☐	Clinical data
☒	☐	Dual use research of concern
☒	☐	Plants

Methods

n/a	Involved in the study	
☒	☐	ChIP-seq
☒	☐	Flow cytometry
☒	☐	MRI-based neuroimaging

Eukaryotic cell lines

Policy information about cell lines and Sex and Gender in Research

Cell line source(s)	SK-MEL-28 (ATCC HTB-72)
Authentication	The cells have not been authenticated.
Mycoplasma contamination	Cell lines were not tested for mycoplasma contamination but no indication of contamination was observed.
Commonly misidentified lines (See ICLAC register)	No commonly misidentified cell lines were used.

Plants

Seed stocks	No seed stocks were used.
Novel plant genotypes	No plants were used.
Authentication	No plants or seeds were used.
