# Supplementary material for: Dynamically reconfigurable acoustofluidic metasurface for subwavelength particle manipulation and assembly
Source: Nat Commun. 2025 Jan 15;16:494. doi: 10.1038/s41467-024-55337-0 (PMC11736025; doi:10.1038/s41467-024-55337-0)
Supplement: Supplementary file 8 — Transparent Peer Review file [file 41467_2024_55337_MOESM8_ESM.pdf]

nature portfolio

Peer Review File

Dynamically reconfigurable acoustofluidic metasurface for subwavelength particle manipulation and assembly

Corresponding Author: Professor Utkan Demirci

This file contains all reviewer reports in order by version, followed by all author rebuttals in order by version.

A version of this paper was originally rejected for publication by Nature Communications, however that decision was reconsidered after appeal by the authors.

Version 0:

Reviewer comments:

Reviewer #1

(Remarks to the Author)
The paper introduces an innovative approach for microscale particle manipulation using a dynamically reconfigurable acoustofluidic metasurface, showcasing the ability to control particle positioning with high precision.
The concept introduced in the paper is intriguing; however, additional details on specific aspects would enhance its comprehensiveness.
The paper does not explicitly address the direct impact of membrane dimensions or spacing on evanescent wave characteristics. In fact, no where in the document the authors say what is the spacing between the membranes and how were these parameters chosen?
It's unclear if the analytical calculations of the 10 eigenvalues from Supplementary Note 1 align with the COMSOL simulations depicted in Figure 2a. Clarification on whether there's evidence linking the COMSOL simulations, analytical explanations from Supplementary Note 1, and experimental characterizations would strengthen the paper's validity.
Specifically, incorporating LDV measurements to corroborate the COMSOL simulations in Figure 2a would be beneficial.
Furthermore, the manuscript could benefit from detailing the scaled units used in the figures. Through out the paper, the authors used scaled units which makes comparisons really hard. On a related note, what is TW in Figure 1 and A.U in the other figures?
Can the authors present some data to show this?
“The subwavelength nature of the excited mode becomes apparent as alternate membranes exhibit a 180-degree out-of-phase displacement. This displacement corresponds to a standing wave with a wavelength of 150 µm when excited by a far-field acoustic source of approximately 1 mm wavelength.”
The discrepancy between the simulated (Figure 3a) and experimental (Figure 3b) patterns, as acknowledged by the authors, raises concerns about the experimental validation of the device's ability to manipulate particles using evanescent waves.
While the authors attribute differences to transducer positioning, the absence of direct correlation weakens their argument.
Atleast to my naked eye there is not much correlation between the two figures.
As another mechanism to validate their approach, I would suggest comparing acoustic radiation forces/ gorkov with drag forces in the system corresponding to Figure 4 and correlate the locations where particles are trapped with the gorkov to cross-validate theoretical expectations with experimental data.
Overall, I liked the ideas in the paper but felt that the different results have not been joined-up properly to present a compelling story that their system works in the way they argue.

Reviewer #2

(Remarks to the Author)
In this work, the authors present a “dynamically reconfigurable silicon-based meta surface designed for trapping and precise positioning of particles within fluidic environments” using acoustofluidic principles. The technique uses a combination of acoustic waves generated in the far field and an array of sub-wavelength speakers fabricated in a grid pattern within the active area of the device. The combination of the acoustic pressure and resulting fluid-structure interaction allows the device

to perform a handful of, what the authors describe as “precise” particle manipulation techniques. The authors refer to the platform as a dynamically reconfigurable acoustofluidic metasurface or “DReAM”.

The team uses finite element analysis to: analyze the platform, better understand the physics of the system, and perform various design optimization tasks. The results presented in figure 2 and discussed in the associated text suggest a comprehensive analysis of the system but don’t appear to reveal any broader new insights on acoustofluidic transport.

The particles are relatively large at 10 um but the team does show some general agreement between the simulation results shown in Figure 3a and the patterns in 3b. I note however that this agreement is qualitative in the sense that one can eyeball the match between the two (mostly) but there is no quantitative comparison of accuracy.

Results shown in Figure 4 are good experiments and provide some characteristic insight into the capture capabilities of the device itself but I don’t see that they broaden knowledge of the field.

In summary, this is a solid paper presenting a unique acoustofluidic device that demonstrates the ability to capture relatively large polystyrene beads in a microfluidic device as well as the ability to perform some level of control over that capture and some minor level of individual particle manipulation. While a solid paper and an interesting device – I don’t think it meets the standard for publication in Nature Communications for the following reasons

- The paper presents a unique device but I don’t see that it offers truly unique, quantitively expressible, capabilities beyond other methods of capturing and manipulating particles of this size. I note that the authors provide no detailed comparison and the discussion is just further discussion of the device.

- I don’t see that the analysis presented provides some deeper understanding of acoustofluidics. The analysis is solid but is specific to this device, new and broader insights on particle transport for example would be difficult to disentangle.

- There is not a unique application presented that results from the capabilities demonstrated. For example if the authors could show that with this new device they were able to perform bioanalytical capability “x” better than how it is done now with state-of-the-art method “y” that would be impressive. What is presented here is largely just characterization with an engineering target.

Version 1:

Reviewer comments:

Reviewer #1

(Remarks to the Author)
I thank the authors for the detailed response and the revised manuscript.
I feel that this revised version is a much stronger document and I would support its acceptance.

Reviewer #2

(Remarks to the Author)
I think the reviewers have addressed most of my comments. The modifications to Figure 4 demonstrate an improved push towards what could be a novel application. I'm OK with publication if all other reviewers are as well - subject to the point below.

In one of the responses, the others state that they have run a Pearson’s correlation coefficient test quantifying the accuracy of their simulations vs experiment. They report values of 0.54, 0.34, 0.59, and 0.77 and state that this represents "high accuracy". My understanding of how such a test works is that these would be mostly "moderate to weak accuracy" (paticularly the first 3). This should be clarified.

Open Access This Peer Review File is licensed under a Creative Commons Attribution 4.0 International License, which permits use, sharing, adaptation, distribution and reproduction in any medium or format, as long as you give appropriate credit to the original author(s) and the source, provide a link to the Creative Commons license, and indicate if changes were made.
In cases where reviewers are anonymous, credit should be given to 'Anonymous Referee' and the source.
The images or other third party material in this Peer Review File are included in the article’s Creative Commons license, unless indicated otherwise in a credit line to the material. If material is not included in the article’s Creative Commons license and your intended use is not permitted by statutory regulation or exceeds the permitted use, you will need to obtain permission directly from the copyright holder.
To view a copy of this license, visit https://creativecommons.org/licenses/by/4.0/

Detailed Response to Reviewers

We greatly appreciate all the reviewer comments that led to new experiments, data and analysis. We closely followed them and overall significantly improved the paper. All additions to the manuscript have been highlighted in yellow.

Reviewer #1

We greatly appreciate Reviewer 1's feedback highlighting the innovative and intriguing aspects of our paper. **We have now included additional supporting data and a more detailed explanation of some of the experimental results** that addresses the issues raised by reviewer 1.

The paper does not explicitly address the direct impact of membrane dimensions or spacing on evanescent wave characteristics. In fact, no where in the document the authors say what is the spacing between the membranes and how were these parameters chosen?

The square membranes can be modelled as a thin plate clamped at the boundaries, and as such, parameters such as the elastic modulus, plate thickness and edge length determine its fundamental resonance frequency. Previously it has been found that the array modes or cross talk modes are found in the vicinity of this single membrane resonance frequency [30]. Thus, the choice of membrane dimensions determines the frequency band in which the reconfigurable array modes are seen. We simulate the frequency response of the DReAM array for three different edge lengths, and we find that the array modes exist nearby the resonance frequency of the single membrane. (supplementary figure 9a and 9b).

The spacing between the membranes determines the shortest possible wavelength that can be supported on the array. Geometrically, the shortest wavelength corresponds to the array mode in which neighboring membranes are 180 degrees out of phase. Hence a smaller membrane pitch allows for an evanescent mode with a shorter wavelength, as seen in supplementary figure 9c. In theory, minimizing the membrane dimension and spacing would result in the array being able to support even shorter subwavelength modes on its surface. However, reducing the membrane dimensions also leads to an increase in the resonance frequency, so any advantage gained in shortening the wavelength is lost by requiring a higher actuation frequency. **To reliably fabricate the arrays and operate them in the 1-2 MHz range, we chose membrane dimensions of 70 μm and a spacing of 5 μm.**

The following details are now included in the supplementary methods 1 of the revised manuscript. The membrane dimensions in both the simulation and experiment are provided in supplementary table 1 and 2.

"Selection of design parameters: The effect of array geometric parameters on wave propagation, such as membrane edge length and pitch, were first simulated to determine the target dimensions. A single membrane can be modelled as a thin plate clamped at the boundaries, and as such, plate dimensions such as edge length and thickness determine the fundamental or resonance frequency of the plate. The natural frequency of three square

plates of different dimensions is shown in supplementary figure 9a. It can be seen that the resonance frequency of the plate increases as the edge length is decreased. Previous work on such resonator arrays[30] has shown that the array/cross-talk modes lie in the vicinity of the single membrane resonance frequency. It is presented in the supplementary figure 9b that the choice of membrane dimensions determines the frequency range in which the dispersive behavior of the array can be observed.
The spacing between the membranes determines the shortest possible wavelength that can be supported on the array. Geometrically, the shortest wavelength corresponds to the array mode in which neighboring membranes are 180 degrees out of phase. Hence a smaller membrane pitch allows for an evanescent mode with a shorter wavelength, as seen in supplementary figure 9c. In theory, minimizing the membrane dimension and spacing would result in the array being able to support the shortest subwavelength modes on its surface. However, reducing the membrane dimensions also leads to an increase in the resonance frequency, so any advantage gained in shortening the wavelength is lost by requiring a higher external actuation frequency. In order to reliably fabricate the arrays and operate them in the 1-2 MHz range, we used membranes with dimensions of 70 μmx70 μm and a spacing of 5 μm."

It's unclear if the analytical calculations of the 10 eigenvalues from Supplementary Note 1 align with the COMSOL simulations depicted in Figure 2a. Clarification on whether there's evidence linking the COMSOL simulations, analytical explanations from Supplementary Note 1, and experimental characterizations would strengthen the paper's validity. Specifically, incorporating LDV measurements to corroborate the COMSOL simulations in Figure 2a would be beneficial.

Thank you for this suggestion, we have now calculated the 10 eigenvalues and included them in the supplementary material in table 4. It can be seen that these eigenvalues lie between 1.5 - 2 MHz and show good agreement with the resonance frequencies derived from both the simulation and experiment. It should be noted here that the analytical model assumes each membrane behaves as a piston radiator, makes use of lumped parameter stiffness and mass values and neglects viscous losses, which explains the expected discrepancies between the analytical, simulated and experimental data.

We would also like to point out that LDV measurements were made on the experimental device to corroborate the COMSOL simulations and presented in figure 2g. A comparison of figure 2a and 2g (simulation vs experiment) shows a very good match in terms of the frequency band in which the evanescent array modes are observed. The frequency response in the simulation shows peaks with a larger Q-factor but this can be attributed to the fact the simulation ignores viscous effects and other material and fluid losses.

We have also included the following sentences in the supplementary section to elaborate on the calculation of eigenvalues using the analytical model:

"Solving these equations for a system comprising ten equally spaced membrane resonators submerged in fluid yields ten eigenvalues with their Q-factors (Supplementary table 4). Note that the eigenvalues and Q-factors calculated using the analytical technique closely align

with the COMSOL simulations (fig 2a) and experimental data (fig 2g). The variations between the eigenfrequencies calculated using each of these techniques can be attributed to the fact that the analytical solution uses lumped parameters, assumes acoustic radiation from a baffled piston, and discards the effects of viscous losses. Likewise, the 2D domain simulated in COMSOL is infinitely long in the z-direction and uses plane wave radiation boundary conditions to model wave propagation. These simplifications also tend to overestimate the Q-factor as they fail to capture all the complex acoustic-fluid-structural interactions that occur in the experimental device."

Furthermore, the manuscript could benefit from detailing the scaled units used in the figures. Through out the paper, the authors used scaled units which makes comparisons really hard. On a related note, what is TW in Figure 1 and A.U in the other figures?

Thank you for your comment. We intentionally used scaled units to obtain a qualitative understanding of the physics driving the system. The scaled units allowed us to focus on the trends and relationships between parameters, rather than absolute values.

Many of the panels in figure 2 depict the relationship between acoustical quantities such as pressure, velocity and radiation force with the geometric boundary conditions of the device, which are more meaningfully represented in scaled units. For example, figure 2c represents how the evanescent wave exponentially decays as one moves away from the metasurface, wheras figures 2b and 2d represent how these acoustic parameters vary as a function of boundary conditions. These figures are meant to provide a qualitative understanding of the acoustic-structure interaction in the system. For this reason, we aimed to illustrate the effects of parameter changes on the system's behavior, which is more insightfully presented in normalized units.

TW in figure 1 is short for 'travelling wave' – we have now included this clarification in the manuscript.
Similarly, a.u. refers to 'arbitrary units' – This number is a measure of the number of bright pixels (representing florescence signal emitted by the trapped beads) as measured by the ImageJ software. This has now been noted in the revised manuscript.

Can the authors present some data to show this?
"The subwavelength nature of the excited mode becomes apparent as alternate membranes exhibit a 180-degree out-of-phase displacement. This displacement corresponds to a standing wave with a wavelength of 150 µm when excited by a far-field acoustic source of approximately 1 mm wavelength."

This comment refers to our statement in the simulation section of the paper, regarding the subwavelength nature of the excited mode. To provide context, we analyze the displacement profile of the simulated membrane array at a frequency of 1.48 MHz, which corresponds to a high Q-factor resonance peak that shows up in the frequency analysis (shown in Fig. 2a). The displacement profile of the domain (shown in Fig. 2b and below) shows that adjacent membranes are 180 degrees out of phase with each other. Since the pitch between the simulated membranes is 80 µm, the distance between two points on the

surface having the same displacement phase is 2 x 80 µm = 160 µm (we acknowledge and have corrected a typo where we mistakenly stated this as 150 µm). Furthermore, the excitation frequency of 1.48 MHz roughly corresponds to a wavelength in water of $\lambda = \frac{c}{f} = \frac{1500\ m/s}{1.48\ MHz} \approx 1\ mm$. This mode represents the shortest subwavelength standing wave that the metasurface can support.

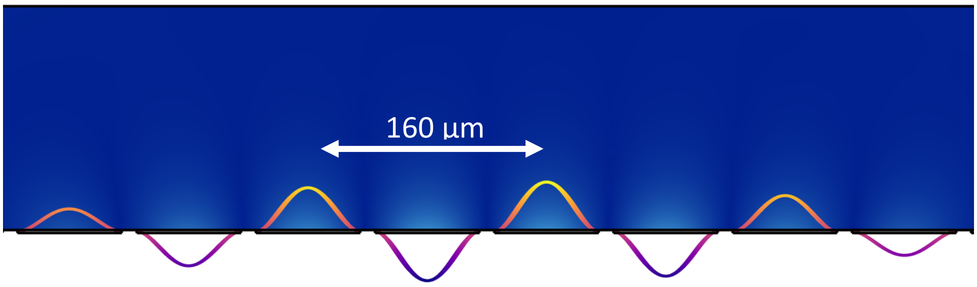

Figure 1: The simulated system shows membranes oscillating out of phase when excited by a far field wave at 1.48 MHz. The wavelength of the evanescent wave on the surface in this case measures 160 µm.

To further validate this, we have included experimental surface plot below, which shows the displacement field above the DReAM array when excited by two far field sources at 1.7 MHz placed above and below the array. The standing wave on the metasurface is a superposition of different modes, where one of the dominant modes excites adjacent membranes 180 degrees out of phase and has a wavelength of approximately 160 µm. The experimental results corroborate the simulation findings, providing strong evidence for the subwavelength nature of the excited mode.

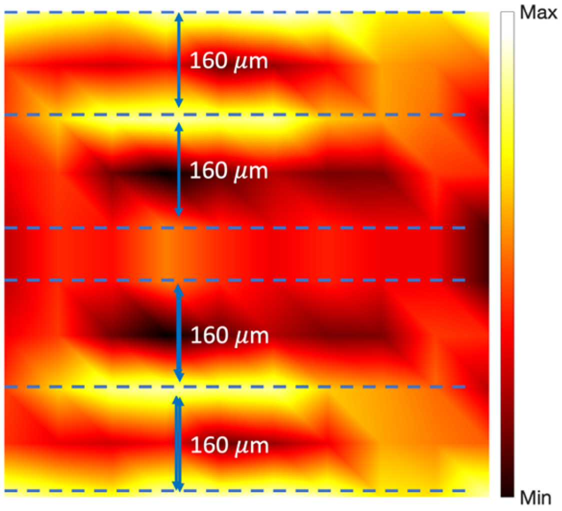

Figure 2: Experimentally measured displacement field at an excitation frequency of 1.7 MHz, with in-phase sources placed above and below the metasurface. The dashed lines represent the contours of the crests of the standing wave. The shortest mode has a wavelength of 160 µm, which is also the minimum wavelength that the surface can support.

The discrepancy between the simulated (Figure 3a) and experimental (Figure 3b) patterns, as acknowledged by the authors, raises concerns about the experimental validation of the device's ability to manipulate particles using evanescent waves. While the authors attribute differences to transducer positioning, the absence of direct correlation weakens

their argument. Atleast to my naked eye there is not much correlation between the two figures.

Thank you for bringing this to our attention. We have revised the format of the figure (see figure 1 below) to show more clearly how the field distribution as measured by the LDV (figure 3a) compares with the bead patterning (figure 3b). We have also included a quantitative comparison as suggested by reviewer 2 that returns a Pearson correlation coefficient as high as 0.77, indicating that the evanescent waves can indeed be used to reconfigurably manipulate the particles above the metasurface.

We also add to discussions of the manuscript clearly and we point out that as noted by reviewer 2, **figure 3a and figure 3b are not meant to correlate perfectly as they are different experiments performed under vastly different boundary conditions – as such they are meant for qualitative comparison**. Figure 3a demonstrates that different field distributions can be obtained by linear superposition of responses obtained from individual sources. The measurements for this were done under free-field conditions. Figure 3b shows that particles in the vicinity of the metasurface can be trapped and arranged in different spatial configurations. This experiment was done by encasing the surface in a microfluidic channel and flowing beads. **Despite the differences in boundary conditions, we find that three of the arrangements in figure 3b are well correlated with the patterns generated in figure 3a.**

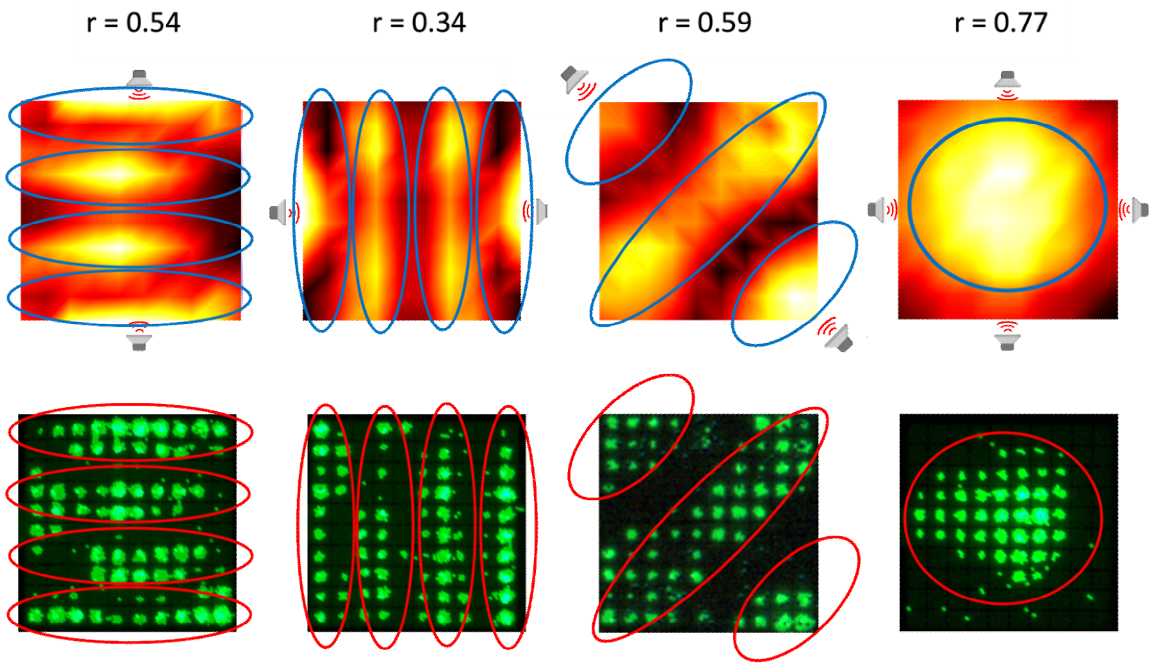

Figure 3: Top panel shows the spatial distribution of standing waves reconstruction from the Green's functions obtained using the LDV. The bottom panel shows how the beads pattern when excited using the same source-frequency combination. Note that the bead experiments were done in an enclosed channel under flow conditions whereas the LDV measurement was done in free-field. Despite this, the particles pattern along the locations estimated by the LDV reconstruction (marked by ovals) with correlations of 0.54,0.34 and 0.77 respectively.

The following sentences have been included in the supplementary material to explain the reason for the differences between figure 3a and 3b in the manuscript.

“Supplementary note 2. Reconstruction of pressure fields over DReAM and particle patterning

The LDV measurement of the metasurface and the subsequent recreation of the fields for different transducer configurations as shown in figure 3a were performed under free-field fluid conditions, i.e., the surface was placed in a large container and filled with water. A single source transducer was placed at each of the 8 positions around the metasurface and provided with an electrical impulse excitation. The response of all the membranes for an excitation from each of the 8 positions was recorded by the LDV. Note that all these measurements were taken on the surface without any boundaries or reflectors nearby. From this entire library of responses, we used linear superposition to reconstruct distinct field distributions that we might obtain for different source combinations and frequencies. The bead patterning shown in figure 3b was achieved under significantly different boundary conditions. A microfluidic channel was created to introduce the beads over the surface in a controllable manner. The top and side walls of the channel immediately introduce reflections and other boundary effects that were not seen by the surface in the free-field LDV measurements. This would naturally cause the field distribution over the metasurface to be different from that generated in free-field conditions. Despite this, we see that bead patterns show qualitative (visual) agreement with the free-field LDV result, i.e., we see distinct patterns with periodicity in the horizontal (1.3 MHz), vertical direction (1.27 MHz) and diagonal directions (1 MHz) and bead concentration predominantly at the center of the surface (720 kHz). Note that the field strength is not the same in each of these cases. For example, the field strength in the 720 kHz mode is significantly weaker than the field in at 1.3 and 1.27 MHz, as it is a more radiative mode. Therefore, the trapping strength is also lower which results in sparse, unpopulated regions at the boundaries of the circular pattern. Quantitative comparison between figure 3a and 3b produced correlation coefficients of 0.54, 0.34, 0.59 and 0.77, implying a moderate to high correlation. Despite a visual match, a lower correlation coefficient can be attributed to the fact the quantitative comparison is between a figure generated from a discrete LDV displacement measurement (10 x 10 matrix) and an optical image (630x630 pixels) showing the patterning of beads on the surface.”

As another mechanism to validate their approach, I would suggest comparing acoustic radiation forces/ gorkov with drag forces in the system corresponding to Figure 4 and correlate the locations where particles are trapped with the gorkov to cross-validate theoretical expectations with experimental data.

Thank you for this suggestion. We have now presented the calculation of the approximate acoustophoretic radiation force acting on the particles in supplementary note 3. We assume that the radiation force and the drag force are approximately equal in magnitude, right before the drag force overcomes the acoustic force and release the trapped particles at flow rates > 200 μL/min (as shown in figure 4f). Based on channel dimensions, particle size and flow rate, we calculate the radiation force at the surface to be approximately 830 pN. The magnitude of this radiation force can be increased or decreased by appropriately adjusting the driving voltage to the source transducers.

“Supplementary note 3. Calculation of Drag Force on a Bead in a Microfluidic Channel

Channel Dimensions and Flow Characteristics
The microfluidic channel has a height (h) of 150 μm, a width (w) of 2.5 mm, and a length (L) of 40 mm. The flow rate (Q) at which the drag force due to flow overcomes the acoustophoretic radiation force on the beads is > 200 μ L/min, which corresponds to an average fluid velocity (v) of:

$$v = \frac{Q}{h \times w} = \frac{3.33 \times 10^{-9}}{150 \times 10^{-6} \times 2.5 \times 10^{-3}} = 0.0088\, m/s$$

Drag Force Calculation
For a spherical bead with a diameter (d) of 10 μm, the drag force (F_d) can be calculated using Stokes' law:

$$F_d = 6\pi\eta r v$$

where η is the fluid viscosity, r is the bead radius, and v is the average fluid velocity.
Using the values:
η = 0.001 Pa· s (viscosity of water at room temperature)
r = 5 μm = 0.000005 m (bead radius)
v = 0.0088 m/s (average fluid velocity)
Now we can assume that right before the trapped beads are overcome by the force of the moving fluid, the radiation force is equal to the drag force. Therefore, the minimum acoustophoretic radiation force acting on the particles cab be estimated as:

$$\mathbf{F_{rad} \approx 6 \times 3.14 \times 0.001 \times 0.000005 \times 0.0088 \approx 829\, pN}$$ “

Moreover, the simulation results shown in Figure 2b present that the radiation forces are most pronounced at the surface of the array, with vectors pointing toward the membranes. Consistent with these findings, particle tracing simulations predict that polystyrene beads will be attracted to and accumulate on the membranes. This behavior is experimentally confirmed by microscopic images showing beads collecting on the membranes, while the rigid areas between membranes remain bead-free.

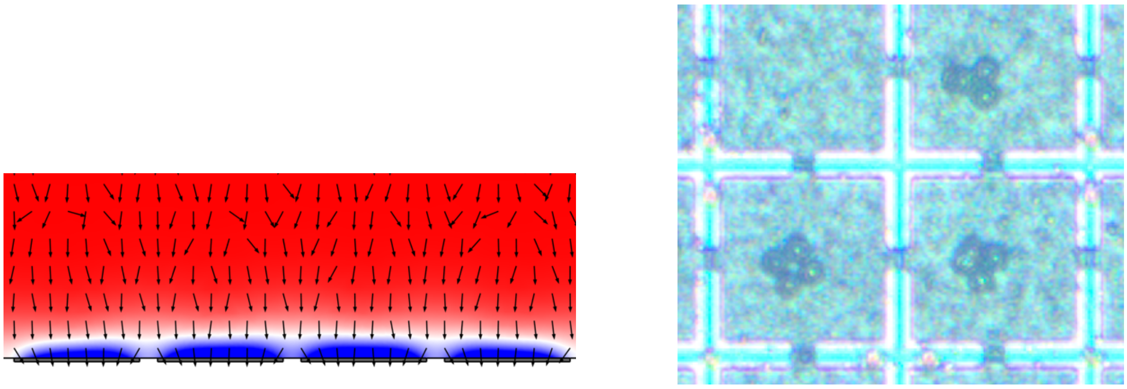

Figure 4: Left pane depicts the simulation showing that the acoustophoretic radiation force points toward the membranes. Right figure is a brightfield microscopic image showing the capture of beads on the metasurface. It can be seen that the beads tend to accumulate on the membranes.

Reviewer #2

We thank the reviewer for the detailed feedback. While they find the paper '**solid**' and the device '**interesting**', Reviewer 2 raised three major concerns regarding 1) the novelty of the approach, 2) the broader impact of the analysis and 3) the unique applications resulting from the capabilities of the device. Each of these concerns is briefly clarified as below, with detailed explanations and supporting evidence presented subsequently in this document:

1. **Novelty** - Our work on reconfigurable acoustofluidic metasurfaces is novel, has unique applications and advances the broader field of acoustofluidics. This work is the first demonstration wherein **reconfigurable** standing waves generated on a **passive membrane-based metasurface**, are used to manipulate microscale particles in static and flow conditions at **subwavelength** resolutions ($\approx \lambda/100$). Our approach has the advantage of a) Lower operating frequency (1- 2 MHz), b) dynamic reconfigurability, c) superior resolution (15 μm) and d) higher throughput (>100 μL) compared to other state-of-art acoustofluidic platforms. Each of these aspects is quantitatively presented in table 1. We have highlighted all these aspects in our revised manuscript.
2. **Broad impact** -The modelling and analysis presented in this paper can be applied more generally to the broader class of metasurfaces in acoustofluidics. The tools developed by us can be used to predict how design parameters such as spacing and resonator stiffness affect the actuation frequencies and wavefield distribution while designing new reconfigurable metasurfaces, which do not currently exist. The analysis also reveals new approaches for selective trapping and release of particles based on their acoustic properties. These impacts are discussed in detail in the attachment and have been incorporated into our revision.
3. **Application** – We have demonstrated that our platform can efficiently concentrate different types of cells from solutions with low initial concentrations to achieve higher concentrations in a reduced final volume (<100 μL). This process aims to enhance the sensitivity and effectiveness of biomarker detection and analysis. Currently, enrichment of such small volumes is very challenging in benchtop approaches such as centrifugation or filtration. We also discuss how our platform can be extended to selectively trap and release flowing particles from specific locations on the metasurface, which could potentially enable to capture, identification and selective release of target cells from a heterogenous population. These results support the broad applicability and wide impact of our presented innovative tool and approach.

The detailed responses to Reviewer 2's comments are as follows:

1. The team uses finite element analysis to: analyze the platform, better understand the physics of the system, and perform various design optimization tasks. The results presented in figure 2 and discussed in the associated text suggest a comprehensive analysis of the system but don't appear to reveal any broader new insights on acoustofluidic transport.

We appreciate the reviewer's acknowledgment of the comprehensive nature of our analysis. As our work represents the **first demonstration** of exploiting acoustic coupling in membrane

resonators to generate reconfigurable acoustic fields for fluidic particle manipulation, we dedicated significant effort to understand and model the underlying mechanisms that govern these interactions. Although our analysis primarily centers on the device outlined in our paper, the conclusions and insights derived from this examination hold unprecedented implications for the broader field of acoustofluidics. We have now added extensive discussions outlining the wider implications of our findings, particularly regarding:

1. Advancing reconfigurable acoustofluidic metamaterials: Our analysis unveils theory and mechanisms that can advance the development of a new class of reconfigurable acoustofluidic metamaterials, which do not currently exist. By demonstrating that surfaces comprising periodic, resonating membranes can support multiple spatially distinct subwavelength wavefields within specific frequency bands, our findings extend to the broader category of locally-resonant acoustofluidic metasurfaces. While previous studies have focused on surfaces with rigid holes or inclusions supporting static wavefields at fixed frequencies, our analysis can be used to uncover additional array modes enabling dynamic reconfigurability in these currently static configurations. Furthermore, the design principles derived from our analysis can guide the creation of new type of reconfigurable metasurfaces with diverse geometries and capabilities. For instance, our analysis reveals that the frequency band where we see acoustic cross-talk is strongly dependent of the natural frequency of the resonating element. The use of compliant membranes as unit resonators in DReAM allows us to exploit its dispersive properties in the low MHz frequency range whereas more traditional acoustofluidic metasurfaces that use rigid inclusions or stiff plates, typically operate at higher frequencies. This information can be used to design metasurfaces that employ soft or compliant materials (such as polymers) as resonators that could potentially allow us access to deep-subwavelength manipulation at submicron scales, circumventing the need for sophisticated interdigital transducers and complex electronics.

2. **Introducing a Complementary Approach to Acoustofluidic Particle Sorting and Enrichment:** Our analysis introduces a novel, complementary approach to acoustofluidic particle sorting and enrichment**. We found and presented for the first time that the evanescent wavefield generated over the metasurface interacts differently with particles possessing distinct acoustic properties.** Particles exhibiting positive contrast factors are attracted to the surface, where they can be isolated from the fluid, while particles with negative contrast factors are repelled from the surface and continue to flow. This contrasts with conventional standing wave approaches, where particles are sorted towards the nodes and antinodes of the standing wave. The ability to selectively attract and repel particles based on their acoustic properties opens a new avenue for sorting and further enrichment in acoustofluidic applications.

The following text has been added in the results and discussion to highlight the significance of our findings:

"**In results**: The simulation and experimental results underscore the unique capabilities of the DReAM array, demonstrating its ability to support multiple dynamically reconfigurable evanescent standing wave modes. These results also provide a broad framework that can be applied to the design and realization of reconfigurable passive acoustic metasurfaces, and

additionally, introduce an innovative approach to spatially localized particle trapping and manipulation at subwavelength scales."

"**In discussion:** Our results provide a foundation for exploring the potential of reconfigurable, locally resonant acoustofluidic metasurfaces, enabling the development of application-driven designs. Simulations reveal remarkable flexibility in the design and realization of these metasurfaces, allowing for tunability of both the strength and spatial distribution of the generated fields. This adaptability is achieved by controlling parameters such as membrane stiffness and resonator arrangement, enabling customization for specific applications."

The particles are relatively large at 10 um but the team does show some general agreement between the simulation results shown in Figure 3a and the patterns in 3b. I note however that this agreement is qualitative in the sense that one can eyeball the match between the two (mostly) but there is no quantitative comparison of accuracy.

Thank you for this comment that allows us to better present our findings. As rightly noted, figures 3a and 3b depict two separate experiments conducted under different boundary conditions and are meant for qualitative comparison. In order to quantify the similarity as suggested by the reviewer, we ran a Pearson's correlation coefficient test. We found that the four images (see figure 1 of response letter) returned a coefficient of 0.54, 0.34, 0.59 and 0.77, implying moderate to high correlation. Despite a visual match, a lower correlation coefficient can be attributed to the fact the quantitative comparison is between a figure generated from a discrete LDV displacement measurement (10 x 10 matrix) and an optical image (630x630 pixels) showing the patterning of beads on the surface.

In addition to a detailed note in the supplementary section highlighting the correlation and the reasons for the differences, we have provided revised figures in the manuscript that better highlights the qualitative agreement between these two experiments.

"**Supplementary note 2. Reconstruction of pressure fields over DReAM and particle patterning**

The LDV measurement of the metasurface and the subsequent recreation of the fields for different transducer configurations as shown in figure 3a were done under free-field fluid conditions i.e. the surface was placed in a large container and filled with water. A single source transducer was placed at each of the 8 positions around the metasurface (supplementary figure 2) and provided with an electrical impulse excitation. The response of all the membranes for an excitation from each of the 8 positions was recorded by the LDV. Note that all these measurements were made on the surface without any boundaries or reflectors nearby. From this entire library of responses, we used linear superposition to reconstruct distinct field distributions that we might obtain for different source combinations and frequencies.

The bead patterning shown in figure 3b was achieved under significantly different boundary conditions. A microfluidic channel was created to introduce the beads over the surface in a controllable manner. The top and side walls of the channel immediately introduce reflections and other boundary effects that were not seen by the surface in the free-field LDV measurements. This would naturally cause the field distribution over the metasurface

to be different from that generated in free-field conditions. Despite this, we see that bead patterns show qualitative (visual) agreement with the free-field LDV result, i.e., we see distinct patterns with periodicity in the horizontal (1.3 MHz), vertical direction (1.27 MHz) and diagonal directions (1 MHz) and bead concentration predominantly at the center of the surface (720 kHz). Note that the field strength is not the same in each of these cases. For example, the field strength in the 720 kHz mode is significantly weaker than the field in at 1.3 and 1.27 MHz, as it is a more radiative mode. Therefore, the trapping strength is also lower which results in sparse, unpopulated regions at the boundaries of the circular pattern.

Quantitative comparison between figure 3a and 3b are produced by calculating the correlation coefficient between corresponding LDV maps and bead patterns. The optical images in figure 3b are first converted into a 10 x 10 grid, where each grid square is assigned an average intensity value of the occupying pixels. The correlation is the calculated using the corr2 function in MATLAB. We obtain correlation coefficients of 0.54, 0.34, 0.59 and 0.77, implying a moderate to high correlation. Despite a visual match, a lower correlation coefficient can be attributed to the fact the quantitative comparison is between a figure generated from a discrete LDV displacement measurement (10 x 10 matrix) and an optical image (630x630 pixels) showing the patterning of beads on the surface. Furthermore, any variations in phase between the LDV measurements and bead patterning would strongly impact the calculated correlation coefficient."

Results shown in Figure 4 are good experiments and provide some characteristic insight into the capture capabilities of the device itself but I don't see that they broaden knowledge of the field.

Conventional acoustofluidic systems require a hard wall or a pair of transducers to generate standing waves axially, along the direction of flow, to trap moving particles using radiation force [35]. Our approach allows us to generate forcefields both parallel and perpendicular to the direction of flow, using a single pair of transducers placed perpendicular to the direction of flow. This technical innovation broadens the knowledge of the field as it allows us to trap and release moving particles in a localized two-dimensional space without the need for hard reflectors or multiple pairs of transducers.

Based on the suggestion from the reviewer, we have now demonstrated how this feature of DReAM can be applied more broadly to the field of bioanalytics. We added a unique application to the paper showing that the metasurface can be used to enrich different cell types occurring in low concentrations in solutions. We also release the enriched cells and test confirm their viability downstream, highlighting the biocompatible nature of our system. This is particularly beneficial in applications like urinalysis, where samples often require initial enrichment using benchtop methods (e.g., centrifugation) to concentrate cells above the detection limit of microscopy.

Furthermore, we've added to the discussion to explore the broader impact of this technology. This includes the potential for sequential surface arrangements, where particles of varying sizes or acoustic properties are trapped or manipulated in distinct regions of the fluidic channel. Additionally, individual membranes can be electrically addressed to trap or release cells from specific locations. This capability enables the selective capture,

identification, and release of target cells within heterogeneous populations, facilitating high-throughput bioanalytical applications such as sensitive detection and screening of specific cell types.

In conclusion, the localized trapping abilities of our system, as described in Figure 4, open the door for innovative approaches to **isolating and enriching biologics with high throughput**, using **off-the-shelf piezoelectric transducers** and without requiring multiple pairs of high-frequency interdigital transducers.. These additions to the results and discussion sections demonstrate the broader impact of our technology, highlighting its potential to transform bioanalytical applications.

"From results:
Building on the platform's ability to trap microscale particles, we demonstrate its capability to enrich biological material from low-concentration solutions. This addresses a significant challenge in the field, where traditional benchtop enrichment methods like filtration or centrifugation are often inadequate for efficiently processing small sample volumes or fragile cells. To evaluate the platform's performance, we passed three different concentrations of 2 ml white blood cell (WBC) solutions through the microfluidic channel at a flow rate of 50 µL/min for 5 minutes. In each case, the cells were trapped and enriched at various points on the 20x20 array when the acoustics was turned on (figure 4g), with the number of trapped cells increasing proportionally with the initial concentration. However, even at the lowest concentration (1,000 cells/mL), a notable 100 ± 24 cells were trapped within the observable array volume (0.34 µL), corresponding to a 294-fold enrichment within this localized region (see methods). Figure 4h further illustrates the impact of acoustic trapping on cell enrichment, showing a marked increase in the number of captured cells over time, with an enhancement in the fluorescence intensity within the array when the acoustic field is applied. As a result, this enables rapid observation of rare cells, even when samples fall below the threshold for microscopic detection, thereby enhancing diagnostic capabilities[37,38]. Additionally, we successfully applied the system to capture SK-MEL-28, a skin cancer cell-line, showcasing its versatility in handling diverse cell types (supplementary figure 4d). Subsequently, we released the trapped SK-MEL-28 cells and assessed their viability, finding negligible effects on cell health following acoustic exposure (supplementary figure 4e). This confirms that the cells remain suitable for downstream analytical processing. These results collectively demonstrate the platform's ability to locally trap and visualize particles and cells on a grid without surface modifications or sheath flows, thus offering a straightforward approach to enrichment and analysis of biological samples at high throughputs."

"From discussion:
Additionally, the planar profile of DReAM facilitates seamless integration with off-the-shelf transducers and microfluidic channels, allowing for capture of microscale particles in flowing conditions. We exploit this feature to gently trap and enrich different cell types from low-concentration solutions, offering a complementary method to centrifugation or other benchtop techniques, especially when handling low-volume samples.
Future developments could include individually addressable electrical control of each membrane, allowing for precise targeting and release of particles to and from specific locations on the metasurface. This capability could enable the capture, identification and selective release of target cells within heterogenous populations, paving the way for high-

throughput bioanalytical applications, including sensitive detection and screening of specific cell types. Integration with optical techniques could expand the system's analytical capabilities, enabling multimodal characterization trapped targets. "

In summary, this is a solid paper presenting a unique acoustofluidic device that demonstrates the ability to capture relatively large polystyrene beads in a microfluidic device as well as the ability to perform some level of control over that capture and some minor level of individual particle manipulation. While a solid paper and an interesting device – I don't think it meets the standard for publication in Nature Communications for the following reasons

- The paper presents a unique device but I don't see that it offers truly unique, quantitively expressible, capabilities beyond other methods of capturing and manipulating particles of this size. I note that the authors provide no detailed comparison and the discussion is just further discussion of the device.

Our work introduces, **for the first time**, **a groundbreaking method** for achieving spatial reconfigurability of the standing acoustic wavefield by manipulating the medium of acoustic wave propagation. Prior work on wavefield reconfiguration in acoustofluidics relied on either manipulating the interference of waves via waveform modulation [26,27] or employing active electronics to reconfigure a lens or hologram [25]. In each of these prior cases, the control over the wavefield is primarily mediated at the acoustic source, rather than the medium through which the waves propagate. While previous studies have utilized surfaces with subwavelength features to locally modify acoustic fields, these fields remained static or had limited manipulating capability [24]. In contrast, we present an innovative approach to wave control wherin the spatial distribution of the acoustic wavefield is governed by the interaction of acoustic waves with a membrane metasurface (propagation medium). **This unique characteristic and finding** allows us and others in the field broadly to exploit tunable fields to dynamically trap and manipulate particles in fluid at subwavelength scales, paving the way for novel unprecedented manipulation capabilities in acoustofluidics with wide applications that would not have been enabled otherwise.

The distinguishing capabilities of our device when compared to other comparable state-of-the-art acoustofluidic manipulations are:

1. In our device, standing wavefields are generated and modified by manipulating the acoustic impedance of the fluid medium. In comparison, other methods rely on the interference of acoustic waves or reflection from boundaries.
2. We can manipulate particles with a resolution of approximately 15 μm using a source frequency near 1 MHz (1.5 mm wavelength).
3. We can re-configure the standing wavefield over a metasurface for the very first time by simply tuning the frequency and phase of the source(s).
4. Our device can trap particles with high throughput at flowrates >100 μL/min.

The following lines have been included in the discussion:

"Reconfigurable acoustic wavefields on a passive metasurface are leveraged to achieve precise manipulation of microscale particles in both static and flowing conditions at

subwavelength resolutions. Current approaches for dynamically modifying wavefields in acoustofluidics rely on either tailoring waveforms to manipulate interference or using phased arrays, with control primarily originating from the acoustic source. In contrast, DReAM leverages acoustic coupling between vibrating membranes to locally modulate the acoustic impedance of the surrounding fluid, offering an innovative strategy for real-time control over the amplitude and phase distribution of the wavefield above the metasurface. This enables particle trapping and arrangement with enhanced resolution (≈15 μm), increased throughput (>100 μL/min) and reduced operating frequencies (< 2 MHz), improving upon existing state-of-art acoustic manipulation platforms[24,26,39,40] (supplementary table 5).“

The following table (now included in the supplementary material) **quantitatively** summarizes the abilities of our device in comparison to similar state-of-the-art acoustofluidic systems manipulating particles in a similar size range (1 – 10 μm)

Table 1.

Published work	Operating frequency	Resolution	Reconfigurability	Mechanism	Sub-wavelength capability	Throughput
Our work (2024)	1 - 2 MHz	15 μm	Yes	metasurface	Yes	200 μl/min
Tayebi, 2020 [24]	50 – 200 MHz	1 μm	No	metasurface	Yes	1 μL/min
Yang, 2022 [26]	40 - 80 MHz	9 μm	Yes	Standing SAW	Yes	No flow
Ma, 2019 [40]	5 MHz	N/A	No	Holography	No	No flow
Tung, 2019 [39]	3 MHz	50 μm	No	Holography	Yes	No flow

- I don’t see that the analysis presented provides some deeper understanding of acoustofluidics. The analysis is solid but is specific to this device, new and broader insights on particle transport for example would be difficult to disentangle.

Our work is presenting how acoustic-structure interactions in a passive metasurface can be used to generate reconfigurable pressure fields in a fluidic environment. The analysis and simulations explain how this unique system works and therefore they clearly bring broader insights into a new operation mode of membrane-based metasurfaces which has not been demonstrated before. As mentioned in our earlier response to reviewer 2, our in-depth analysis of the device presented in the paper leads to deeper and broader insights into a class of previously unexplored, **reconfigurable acoustofluidic metamaterials** and other unprecedented complementary approaches to **particle sorting and enrichment**.

Our work introduces a new approach to particle manipulation in acoustofluidic media by utilizing a fully passive metasurface. The unique nature of our demonstration represents the first instance of an acoustic metasurface that can be employed to generate reconfigurable

evanescent wavefields at subwavelength scales. Our analysis explores the acoustic-structure interactions between resonators as well as the modal properties of the metasurface, that forms the basis for these reconfigurable wavefields. The principles and mechanisms revealed in our research lay the groundwork for a new class of reconfigurable acoustofluidic metamaterials. These advancements offer the potential to significantly enhance subwavelength particle manipulation capabilities, bringing valuable new insights to the field.

Our analysis also revealed some exciting new ways in which particles can be sorted using acoustics. Simulations showed us that particles with different acoustic properties can be selectively attracted or repelled from the metasurface. Such a mechanism contrasts with conventional standing wave approaches wherein a particle either moves toward a node or antinode, depending on its properties. Thus, we present a computational model as a design tool that can be broadly applied to develop new acoustofluidic systems that offer an innovative and complementary approach to particle manipulation, isolation and enrichment.

The following discussions have been added to the manuscript:

"Our results establish a foundation for further exploring the potential of reconfigurable, locally resonant acoustofluidic metasurfaces, enabling the development of application-driven designs. Simulations reveal remarkable flexibility in the design and realization of these metasurfaces, allowing for tunability of both the strength and spatial distribution of the generated fields. This adaptability is achieved by controlling parameters such as membrane stiffness and resonator arrangement, enabling customization for specific applications. We experimentally confirm this tunability by generating diverse wavefield configurations, which enable precise trapping, patterning, and manipulation of particles at both individual and collective scales in a reversible and dexterous manner. This ability to control and organize matter across multiple scales has significant promise for advancing the understanding of soft matter physics, enabling the creation of precisely controlled spheroids for organoid engineering, and allowing for the manipulation and analysis of individual cells in single-cell studies. Additionally, the planar profile of DReAM facilitates seamless integration with off-the-shelf transducers and microfluidic channels, allowing for capture of microscale particles in flowing conditions. We exploit this feature to gently trap and enrich different cell types from low-concentration solutions, offering a complementary method to centrifugation or other benchtop techniques, especially when handling low-volume samples.

Further optimization of array and channel design will enable particle isolation and enrichment with greater efficiencies and recovery rates. The current geometry is capable of trapping particles down to two microns, but redesigning DReAM with a smaller pitch and more compliant resonators could enable support for shorter wavelengths, potentially enabling nanoscale particle manipulation at low MHz frequencies. The ability of DReAM to selectively attract or repel particles based on their acoustic properties can be exploited to develop complementary sorting and isolation approaches. Future developments could include individually addressable electrical control of each membrane, allowing for precise targeting and release of particles to and from specific locations on the metasurface. This capability could enable the capture, identification and selective release of target cells within heterogenous populations, paving the way for high-throughput bioanalytical applications,

including sensitive detection and screening of specific cell types. Integration with optical techniques could expand the system's analytical capabilities, enabling multimodal characterization trapped targets. Ultimately, the system's versatility and compatibility with commercial transducers offers a cost-effective approach to the manipulation of particulate structures, paving the way for diverse applications ranging from self-assembly to point-of-care diagnostics."

There is not a unique application presented that results from the capabilities demonstrated. For example if the authors could show that with this new device they were able to perform bioanalytical capability "x" better than how it is done now with state-of-the-art method "y" that would be impressive. What is presented here is largely just characterization with an engineering target.

Thanks to the reviewer's suggestion, we have now included data that demonstrates how DReAM can be used to enrich biological cells occurring in low concentrations. Currently, benchtop techniques such as filtration and centrifugation are used to pre-concentrate or enrich cells, however this is typically cumbersome to achieve in low volumes (<100 uL) and can also lead to loss of analyte. We show that white blood cells can be enriched on the metasurface (approx. 300-fold enrichment) from solutions containing as few as 1000 cells/ml. We further demonstrate the system's compatibility with diverse cell types by successfully enriching SK-MEL-28 cell-line and verifying their viability post-release. The ability to enrich and visualize cells in a localized region (1.5 mm x 1.5 mm) of the microfluidic channel without any surface modifications or sheath flows opens new possibilities for identification rare cells and high-throughput screening of specific cell types.

Figure 4 has been modified in the revised manuscript to show these results. The following paragraph has also been included to discuss the results:

"Building on the platform's ability to trap microscale particles, we demonstrate its capability to enrich biological material from low-concentration solutions. This addresses a significant challenge in the field, where traditional benchtop enrichment methods like filtration or centrifugation are often inadequate for efficiently processing small sample volumes or fragile cells. To evaluate the platform's performance, we passed three different concentrations of 2 ml white blood cell (WBC) solutions through the microfluidic channel at a flow rate of 50 µL/min for 5 minutes. In each case, the cells were trapped and enriched at various points on the 20x20 array when the acoustics was turned on (figure 4g), with the number of trapped cells increasing proportionally with the initial concentration. Notably, even at the lowest concentration (1,000 cells/mL), 100 ± 24 cells were found to be trapped within the observable array volume (0.34 µL), corresponding to a 294-fold enrichment within this localized region (see methods). Figure 4h further illustrates the impact of acoustic trapping on cell enrichment, showing a marked increase in the number of captured cells over time, with an enhancement in the fluorescence intensity within the array when the acoustic field is applied. As a result, this enables rapid observation of rare cells, even in samples that fall below the threshold for microscopic detection, thereby enhancing diagnostic capabilities[37,38]. Additionally, we successfully applied the system to capture SK-MEL-28, a skin cancer cell-line, showcasing its versatility in handling diverse cell types

(supplementary figure 4d). After releasing the trapped SK-MEL-28 cells, viability assessments indicated minimal impact on cell health due to acoustic exposure (Supplementary Figure 4e), confirming that the cells remain viable for downstream analytical processing. These results collectively demonstrate the platform's ability to locally trap and visualize cells on a grid without surface modifications or sheath flows, thus offering a straightforward approach to enrichment and analysis of biological samples at high throughputs.”

Response to Reviewers

We thank the reviewers for their valuable critique and comments. The various suggestions and feedback has helped us greatly improve the quality and presentation of our manuscript.

In one of the responses, the others state that they have run a Pearson's correlation coefficient test quantifying the accuracy of their simulations vs experiment. They report values of 0.54, 0.34, 0.59, and 0.77 and state that this represents "high accuracy". My understanding of how such a test works is that these would be mostly "moderate to weak accuracy" (paticularly the first 3). This should be clarified.

We define a correlation coefficient of < 0.3 as weak, >0.3 but <0.7 as moderate and >0.7 as high accuracy. As since all of our correlation coefficients are between 0.3 and 1, we state that we obtain moderate to high degree of match between figure 2a and 2b.

In order to clarify this, we have now added the following sentence to explain the scale.

'We obtain correlation coefficients of 0.54, 0.34, 0.59 and 0.77, implying a moderate to high correlation (based on the scale: < 0.3 - low, 0.3 – 0.7 - moderate and > 0.7 - high).'
